# Gastrodin induces lysosomal biogenesis and autophagy to prevent the formation of foam cells via AMPK‐FoxO1‐TFEB signalling axis

**DOI:** 10.1111/jcmm.16600

**Published:** 2021-05-10

**Authors:** Jun Tao, Ping Yang, Liqiu Xie, Yuwei Pu, Jiazhi Guo, Jianlin Jiao, Lin Sun, Di Lu

**Affiliations:** ^1^ Science and Technology Achievement Incubation Center Kunming Medical University Kunming China; ^2^ Department of Anatomy Faculty of Basic Medical Sciences Kunming Medical University Kunming China; ^3^ Department of Cardiology the Second Affiliated Hospital Kunming Medical University Kunming China

**Keywords:** autophagy, foam cells, gastrodin, lysosomal function, lysosome biogenesis

## Abstract

Abnormal accumulation of lipids and massive deposition of foam cells is a primary event in the pathogenesis of atherosclerosis. Recent studies have demonstrated that autophagy and lysosomal function of atherosclerotic macrophages are impaired, which exacerbates the accumulation of lipid in macrophages and formation of foam cells. Gastrodin, a major active component of Gastrodia elata Bl., has exerted a protective effect on nervous system, but the effect of gastrodin on atherosclerotic vascular disease remains unknown. We aimed to evaluate the effect of gastrodin on autophagy and lysosomal function of foam cells and explored the mechanism underlying gastrodin's effect on the formation of foam cells. In an in vitro foam cell model constructed by incubating macrophages with oxygenized low‐density lipoproteins (ox‐LDL), our results showed that lysosomal function and autophagy of foam cells were compromised. Gastrodin restored lysosomal function and autophagic activity via the induction of lysosomal biogenesis and autophagy. The restoration of lysosomal function and autophagic activity enhanced cholesterol efflux from macrophages, therefore, reducing lipid accumulation and preventing formation of foam cells. AMP‐activated protein kinase (AMPK) was activated by gastrodin to promote phosphorylation and nuclear translocation of forkhead box O1 (FoxO1), subsequently resulting in increased transcription factor EB (TFEB) expression. TFEB was activated by gastrodin to promote lysosomal biogenesis and autophagy. Our study revealed that the effect of gastrodin on foam cell formation and that induction of lysosomal biogenesis and autophagy of foam cells through AMPK‐FoxO1‐TFEB signalling axis may be a novel therapeutic target of atherosclerosis.

## 1 INTRODUCTION

The lysosome is a subcellular organelle generally residing in all animal cells.^1^ It is central to the endocytic, autophagic and phagocytic pathways, degrading cellular debris, damaged organelles and protein aggregates.^2^ Lysosome function may be varied by environmental cues,^3^ age^4^ and diseases^5^ and is linked to the pathogenesis of many neurodegenerative diseases.^6^ Enhanced lysosome biogenesis can promote the clearance of protein aggregates, cholesterol and damaged organelles, which relieves the progress of such lysosome‐associated diseases.^7^, ^8^, ^9^

Autophagy is an evolutionarily conserved cellular catabolic process.^10^ A growing number of studies have shown that it plays a fundamental role in the maintenance of intracellular homeostasis via degradation of waste cytoplasmic contents in lysosomes.^11^ A complete autophagic process consists of two stages: in the initial stage of autophagy, the vesicle nucleates and elongates, forming an autophagosome, which accompanied by engulfment of damaged organelles, protein aggregates or lipid granules.^12^ Subsequently, the autophagosome fuses with the lysosome, transporting the cytoplasm contents to the lysosome for degradation.^12^, ^13^ Autophagy has been reported to participate in several pathological and physiological processes, such as immunity, inflammation and stress resistance.^14^, ^15^ Beyond these aforementioned roles, it is also implicated in the occurrence and development of atherosclerosis.^16^

Atherosclerosis is a progressive and lipid metabolism dysfunction‐derived inflammatory disease.^17^ It is a leading cause of acute cardiovascular events, such as myocardial infarction, stroke and ischaemic heart failure.^18^, ^19^ Growing evidence suggests that macrophages play a critical role in the pathogenesis of atherosclerosis.^20^, ^21^ In the initial stage of atherogenesis, macrophages engulf excessive ox‐LDL or other modified lipoproteins, forming lipid‐rich foam cells, a marker of early atherosclerosis.^22^ The appearance of foam cells exacerbates both the inflammatory process and the formation of unstable plaque structures. Therefore, reducing the formation of foam cells may be a promising target for the treatment of atherosclerosis.^20^ Most recently, several lines of evidence have demonstrated that atherosclerotic macrophages have features of autophagy dysfunction.^9^, ^16^, ^23^ Enhancing autophagic activity markedly decreases the formation of foam cells and improves plaque stability.^16^, ^23^ Previous studies have shown that lysosomal dysfunction in macrophages is responsible for foam cell formation and plaque progress.^5^, ^24^, ^25^ Induction of lysosomal biogenesis by genetic or pharmacological intervention can rescue atherosclerosis‐relevant factors‐induced lysosomal dysfunction.^5^, ^9^ Hence, a therapeutic regimen directly targeting autophagy and lysosomal biogenesis of macrophages is critical for treatment of atherosclerosis.^5^, ^9^, ^26^

Statins are a known lipid‐lowering drug generally used for the prevention and treatment of atherosclerotic cardiovascular diseases, but their clinical translation has remained unsatisfactory.^27^ Therefore, it is essential to seek a new therapeutic avenue for atherosclerotic cardiovascular diseases. In China, the ancient Chinese herb Tianma (Gastrodia elata Bl.) has a very long history of clinical application and has been suggested to be effective on cerebrovascular and cardiovascular diseases, such as stroke, cardiac hypertrophy and hypertension.^28^ Gastrodin is a major bioactive component in Gastrodia elata Bl. and has been reported to have anti‐inflammatory and anti‐oxidative activity.^29^, ^30^ At present, gastrodin is widely used in clinic to treat nervous system diseases, such as neurasthenia, dizziness, epilepsy and vertebral basilar artery insufficiency.^30^ In vivo studies suggest that gastrodin also has the therapeutic potentiality for neurodegenerative diseases, such as Parkinson's disease and Alzheimer's disease.^31^, ^32^ Recent evidence demonstrates that powdered Gastrodia elata Bl. reduces blood lipids and liver lipid accumulation in rats fed with fat emulsion.^33^ Furthermore, a report from Geng et al have demonstrated that gastrodin decreases intracellular lipid accumulation in oleic acid‐stimulated liver cells.^34^ Based on these studies, we speculated that gastrodin may affect the formation of foam cells and exert a protective role against atherosclerosis. However, the effect of gastrodin on foam cell formation remains unknown.

In this study, we investigated whether gastrodin affected the formation of foam cells and the underlying molecular mechanism. Our results demonstrated that foam cells were characterized by lysosomal dysfunction and autophagy deficiency. Furthermore, gastrodin restored lysosomal function and autophagic activity through induced lysosomal biogenesis and enhanced autophagic activity, respectively. The restoration of lysosomal function and autophagic activity inhibited lipid accumulation and inflammation to prevent the foam cell formation. Finally, we showed that gastrodin‐regulated lysosomal biogenesis and autophagy through AMPK‐FoxO1‐TFEB signalling axis.

## 2 MATERIALS AND METHODS

### 2.1 Reagents and antibodies

Bafilomycin A1 (Baf A1) was obtained from MedChem Express (catalog no. HY‐100558, Princeton, USA). Dorsomorphin dihydrochloride (Compound C dihydrochloride) was purchased from MedChem Express (catalog no. HY‐13418, Princeton, USA). Antibodies against p‐AMPK (1:1000; catalog no. AF3423), AMPK (1:1000; catalog no. DF6361), TFEB (1:1000; catalog no. AF7015), IL‐6 (1:1000; catalog no. DF6087) and H3 (1:3000; catalog no. BF9211) were purchased from Affinity Biosciences (OH, USA). LC3A/B (1:1000; catalog no. 4108), IL‐1β (1:1000; catalog no. 12242), GAPDH (1:1000; catalog no. 5174), p‐FoxO1/FoxO3a (1:1000; catalog no. 9464) and FOXO1 (1:1000; catalog no. 2880) were purchased from Cell Signaling Technology (Danvers, MA, USA). LAMP1 (1:1000; catalog no. A16894), ABCA1 (1:1000; catalog no. A16337), IL‐18 (1:1000; catalog no. A16737), Beclin 1 (1:1000; catalog no. A11761), CD36 (1:1000; catalog no. A5792), TNF‐α (1:1000; catalog no. A11534) and CTSD (1:1000; catalog no. A13292) were purchased from ABclonal Technology (Wuhan, China). SQSTM 1/p62 (1:1000; catalog no. ab240635) was purchased from Abcam (Cambridge, the United Kingdom). The secondary antibodies HRP‐conjugated anti‐mouse IgG and HRP‐conjugated anti‐rabbit IgG were purchased from Affinity Biosciences (OH, USA).

### 2.2 Cell culture and induction of foam cells

The murine macrophage RAW264.7 cell line was provided by Dr Zhongshan Yang (Yunnan University of Traditional Chinese Medicine) and cultured in DMEM medium (Corning) containing 10% FBS (HyClone, Logan), 100 U/mL penicillin and 100 μg/mL streptomycin. Cells were incubated with 50 mg/L ox‐LDL (Yiyuan Biotech) for 24 h to induce the formation of foam cells.

### 2.3 Quantitative reverse transcription‐polymerase chain reaction (RT‐PCR)

Total RNAs were extracted from cells with TRIzol Reagent (Invitrogen). Random‐primed cDNAs were generated by reverse transcription of the total RNA samples with QuantScript RT Kit (TAKARA). A quantitative real‐time PCR was performed using SYBR Premix Ex Taq (TAKARA) on a Roche LightCycler 480 System (Roche Applied Science). β‐actin was used as an internal control. The primer sequences used for PCR are listed in table S1.

### 2.4 Lentivirus‐mediated targeted disruption of TFEB or FoxO1

The knockdown of TFEB or FoxO1 in macrophages was obtained through lentivirus‐mediated targeted disruption according to the manufacturer's protocol. Briefly, the cells were plated in 6‐well plates. After cultured overnight, the medium was changed to Opti‐MEM (Invitrogen) containing lentivirus, incubating for 12h. Scrambled shRNA was used as a negative control. The RNA oligos used for shRNAs are listed in table S2 (GenePharma).

### 2.5 Establishment of cell lines stably expressing mRFP‐GFP‐LC3

An mRFP‐GFP tandem fluorescent‐tagged LC3 (mRFP‐GFP‐LC3) lentivirus (GenePharma) was used to monitor autophagic flux as previously reported.^35^ Briefly, macrophages were seeded in a 6‐well plate. After 24 h, the medium was changed to Opti‐MEM (Invitrogen) with lentivirus, incubating for 12 h. Then, the cells were selected with growth medium supplemented with 4μg/mL puromycin to obtain positive cell lines stably expressing mRFP‐GFP‐LC3. The co‐localized yellow fluorescence of both GFP and RFP is used as an indicator of autophagosome, and the co‐localized red fluorescence represents the autolysosome.^10^, ^11^, ^35^

### 2.6 Immunofluorescence

Immunofluorescence analysis was conducted as previously described.^36^ Macrophages were seeded onto coverslip in a 24‐well plate. After treatment, cells were washed with PBS, fixed with 4% paraformaldehyde and permeabilized with 0.1% Triton X‐100. After that, cells were blocked with 5% goat serum and then incubated with respective primary antibodies, followed by incubation with the secondary antibodies. Genomic DNA was stained with 4', 6‐diamidino‐2‐phenylindole (DAPI). The fluorescence images were captured using a Zeiss Axioskop 2 plus fluorescence microscope (Carl Zeiss). The fluorescence intensity was analysed using ImageJ software (NIH).

### 2.7 Western blotting analysis

Cell lysates were prepared using RIPA buffer (Beyotime Institute of Biotechnology). Protein concentration was determined by BCA assay (Beyotime Institute of Biotechnology). Equal amounts of total protein were loaded on SDS polyacrylamide gel and then transferred to PVDF membrane (Millipore, MMAS, USA). The membrane was blocked with skim milk and incubated with the primary antibodies, followed by incubation with the secondary antibodies. The protein bands were visualized using an ECL Western blotting detection kit (Millipore, MMAS, USA). Protein intensity was analysed using ImageJ software (NIH).

### 2.8 Oil Red O staining

To determine lipid accumulation and foam cell formation, Oil Red O (ORO) staining was executed as previously described.^37^ Macrophages were fixed with 4% paraformaldehyde and then dehydrated with 60% isopropanol. After that, the cells were stained using an Oil Red O stain kit (Solarbio Life Science). Next, the cells were stained using haematoxylin dye to counterstain the cell nuclei. Images were observed by light microscope.

### 2.9 Estimation of lysosomal biogenesis and intralysosomal pH

Assessment of the lysosomal biogenesis in macrophages was performed as described previously.^38^ After treatment, cells were washed three times with PBS, followed by incubation with LysoTracker (Cell Signaling Technology) for 30 min. The intralysosomal pH in macrophages was estimated using LysoSensor (Dalian Meilun Biotechnology) according to the manufacturer's process. Briefly, cells were washed three times with PBS, followed by incubation with LysoSensor for 30 min. The fluorescence was visualized using a Zeiss Axioskop 2 plus fluorescence microscope (Carl Zeiss). The fluorescence intensity was analysed using ImageJ software (NIH).

### 2.10 Statistical analysis

Data are given as mean ± SD. Statistical analyses were performed using one‐way ANOVA with multiple comparisons and two‐tailed Student's t tests. Value was considered to be statistically significant when *P < .05, **P < .01 and ***P < .001.

## 3 RESULTS

### 3.1 Atherosclerotic macrophages have features of impaired autophagy and dysfunctional lysosomes

**FIGURE 1:**
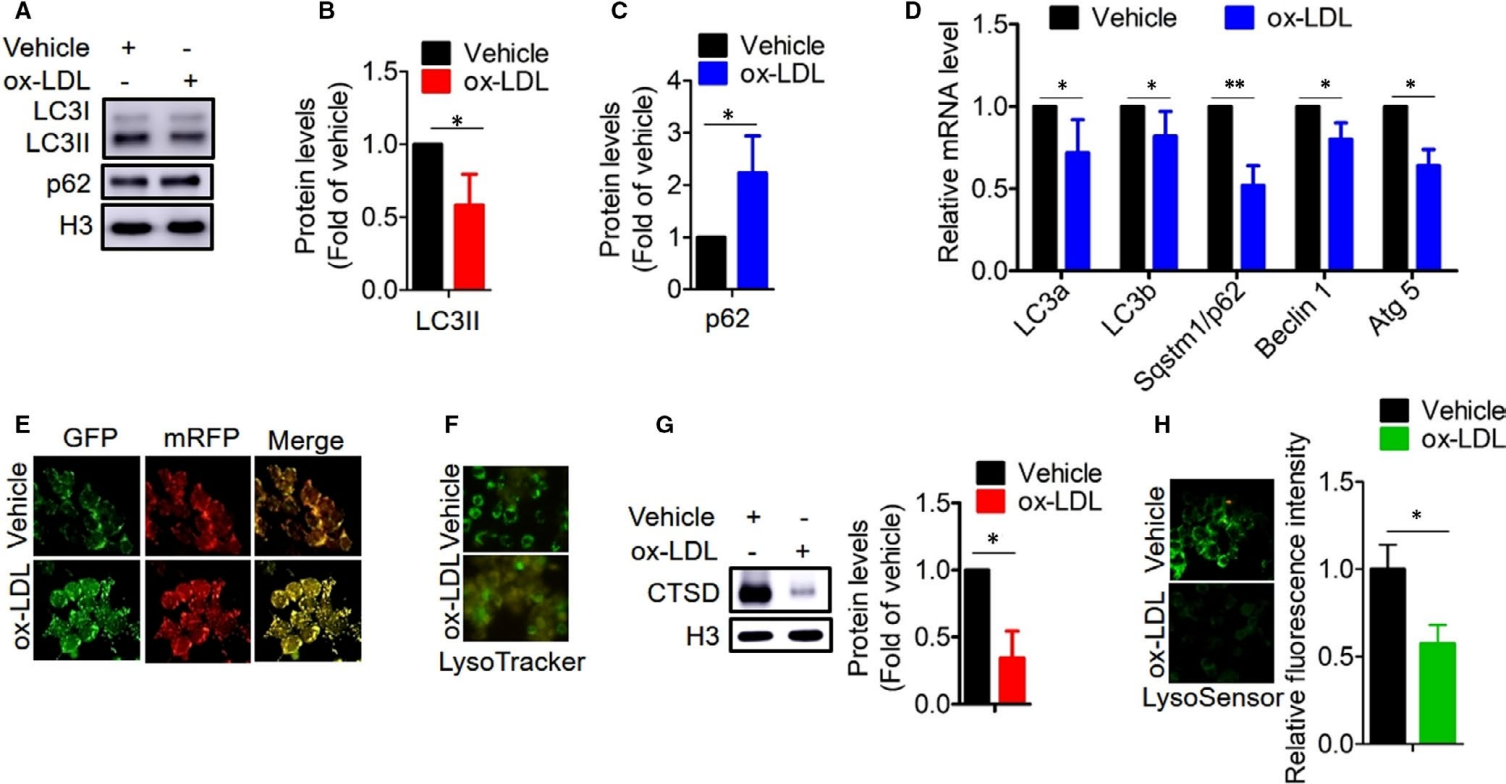
Foam cells are characterized by autophagy deficiency and lysosomal dysfunction. A‐C, Protein levels of LC3I/II and p62 were determined by Western blotting. Representative Western blotting A, and quantitative analysis B and C, displayed a reduced protein level of LC3II and increased protein level of p62. D, The mRNA expression levels of autophagic genes were determined by RT‐PCR. E, Foam cells displayed reduced autolysosomes as shown by the reduced red puncta of mRFP‐GFP‐LC3. F‐H, Measurement of lysosomal biogenesis and function. F, LysoTracker staining for lysosomal biogenesis. G, Protein level of CTSD for lysosomal degradation capability. H, LysoSensor staining for pH. *P < .05; **P < .01. Results are means ± SD of three independent experiments. The value represents fold of vehicle

It has been reported that autophagy plays an important role in the formation of foam cells and plaque complexity.^9^ We first determined autophagic status in atherosclerotic macrophages (referred to as foam cells hereafter). LC3 (an autophagosome coat protein) and SQSTM 1/p62 (a selective autophagy chaperone) are commonly used as autophagy markers.^9^ We found that the enrichment of phosphatydilethanolamine‐LC3 (LC3II) was reduced after exposure to ox‐LDL, whereas the p62 protein level was inversely elevated (Figure 1A‐C). Using RT‐PCR, we found that the mRNA expression levels of autophagic genes were reduced (Figure 1D). To further confirm these results, we introduced a lentivirus harbouring tandem fluorescent mRFP‐GFP‐LC3 into macrophages. In this tandem fluorescent mRFP‐GFP‐LC3 construct, mRFP (monomeric red fluorescent protein) is stable and retains its fluorescence in the acidic environment of lysosomes whereas GFP is sensitive to acidic condition and readily degraded.^39^ As shown in Figure 1E, we observed a reduced number of red puncta in the macrophages treated with ox‐LDL compared to the macrophages treated with vehicle. These results indicate impaired autophagy and interrupted autophagic flux in the foam cells.

As the occurrence of autophagy requires an enhancement in lysosomal function,^1^, ^3^ we in turn evaluated lysosomal function in the foam cells, observing that cell fluorescence under LysoTracker was significantly lower (Figure 1F). Consistent with this result, we tested lysosomal degradation capacity and pH in the foam cells, finding that cathepsin D (CTSD) expression, a major lysosomal endopeptidase required for the degradation of long‐lived proteins,^40^ was markedly decreased, and pH was raised as demonstrated by LysoSensor staining (Figure 1G and 1H), indicating lysosomal dysfunction. Collectively, these results demonstrate that autophagy is deficient and lysosomal function is impaired in the foam cells.

### 3.2 Gastrodin induces lysosomal biogenesis

The phenolic glucoside gastrodin has been reported to exert both anti‐oxidative and anti‐inflammatory effects.^41^ However, whether gastrodin regulates lysosomal biogenesis in cells is unknown. Thus, we explored the effect of gastrodin on lysosomal biogenesis in macrophages. LAMP1 is lysosome‐associated membrane protein 1, commonly used as the indicator of lysosomal biogenesis.^2^, ^42^ Macrophages were treated with different concentration of gastrodin for 24 h, and we found that the expression level of LAMP1 in these cells was increased in a concentration‐dependent manner and that the expression level is highest at the concentration of 20μM. (Figure S1A). To further support this observation, we determined the protein level of LAMP1 at the concentration of 20 μmol\L of gastrodin via Western blotting and immunofluorescence. We found that the expression level of LAMP1 was also increased (Figure S1B and S1C). Using LysoTracker stain, the result also showed an increased fluorescence intensity in the macrophages treated with 20μmol\L of gastrodin (Figure S1D). Consistent with these results, we found that gastrodin significantly increased the mRNA levels of lysosomal biogenesis‐related genes (Figure S1E). Taken together, these results suggest that gastrodin promotes the biogenesis of a functionally normal lysosome.

### 3.3 Gastrodin rescues lysosomal dysfunction and autophagy deficiency in the foam cells

**FIGURE 2:**
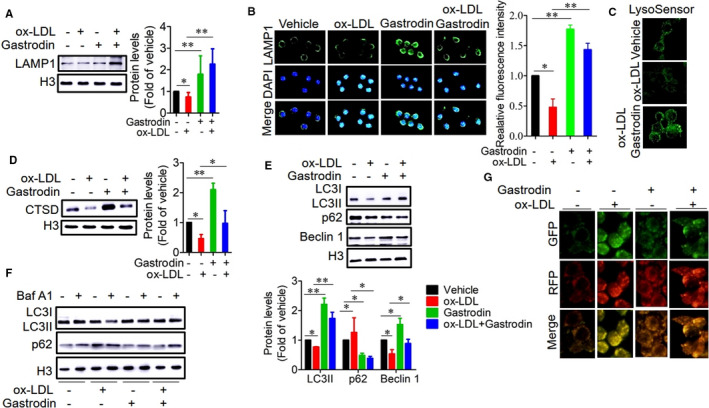
Gastrodin rescues lysosomal dysfunction and autophagy deficiency in foam cells. A and B, Gastrodin‐induced lysosomal biogenesis in the foam cells. Protein level of LAMP1 was determined by Western blotting A, and immunofluorescence B. C and D, Gastrodin rescued the lysosomal dysfunction of foam cells. Gastrodin enhanced LysoSensor staining C, and increased the protein level of CTSD D. E‐G, Gastrodin rescued the impaired autophagy of foam cells. Expression levels of autophagic proteins (LC3I/II, p62 and Beclin1) were analysed by Western blotting in macrophages treated with gastrodin and ox‐LDL in the absence E, and presence F, of Baf A1 (25nM). G, Gastrodin increased the number of autolysosomes in the foam cells as shown by the elevated red puncta of mRFP‐GFP‐LC3. *P < .05; **P < .01. Results are means ± SD of three independent experiments. The value represents fold of vehicle

A recent study has demonstrated that the induction of lysosomal biogenesis can rescue lysosomal dysfunction in atherosclerotic macrophages.^5^ Given our finding that gastrodin induced the biogenesis of a functionally normal lysosome, we examined whether gastrodin rescued lysosomal dysfunction of foam cells via increasing lysosomal biogenesis. We found that the protein level of LAMP1 was remarkably increased in the foam cells in the presence of gastrodin (Figure 2A and 2B), indicating lysosomal biogenesis is induced by gastrodin in the foam cells. Next, we examined the lysosomal pH and the protein level of CTSD and found that gastrodin treatment induced a significant increase in LysoSensor staining and protein level of CTSD in the foam cells (Figure 2C and 2D). These results demonstrate that gastrodin rescues lysosomal dysfunction by inducing lysosomal biogenesis in the foam cells.

Following, we investigated whether gastrodin could rescue the impaired autophagy of foam cells. We observed that gastrodin treatment resulted in a remarkable elevation of the protein levels of LC3II and Beclin 1, a key regulator of the autophagic machinery,^43^ in the foam cells, while p62 protein level was reduced (Figure 2E). After Baf A1 treatment, an inhibitor of lysosomal acidification,^44^ the protein levels of LC3II and p62 were further elevated (Figure 2F). Supporting these results, mRFP‐GFP‐LC3 reporter analysis also showed an increased number of red fluorescent puncta in the foam cells treated with gastrodin (Figure 2G). Collectively, our observations demonstrate that gastrodin rescues lysosomal dysfunction and impaired autophagy in the foam cells.

### 3.4 Gastrodin regulates macrophage cholesterol metabolism to inhibit the formation of foam cells

**FIGURE 3:**
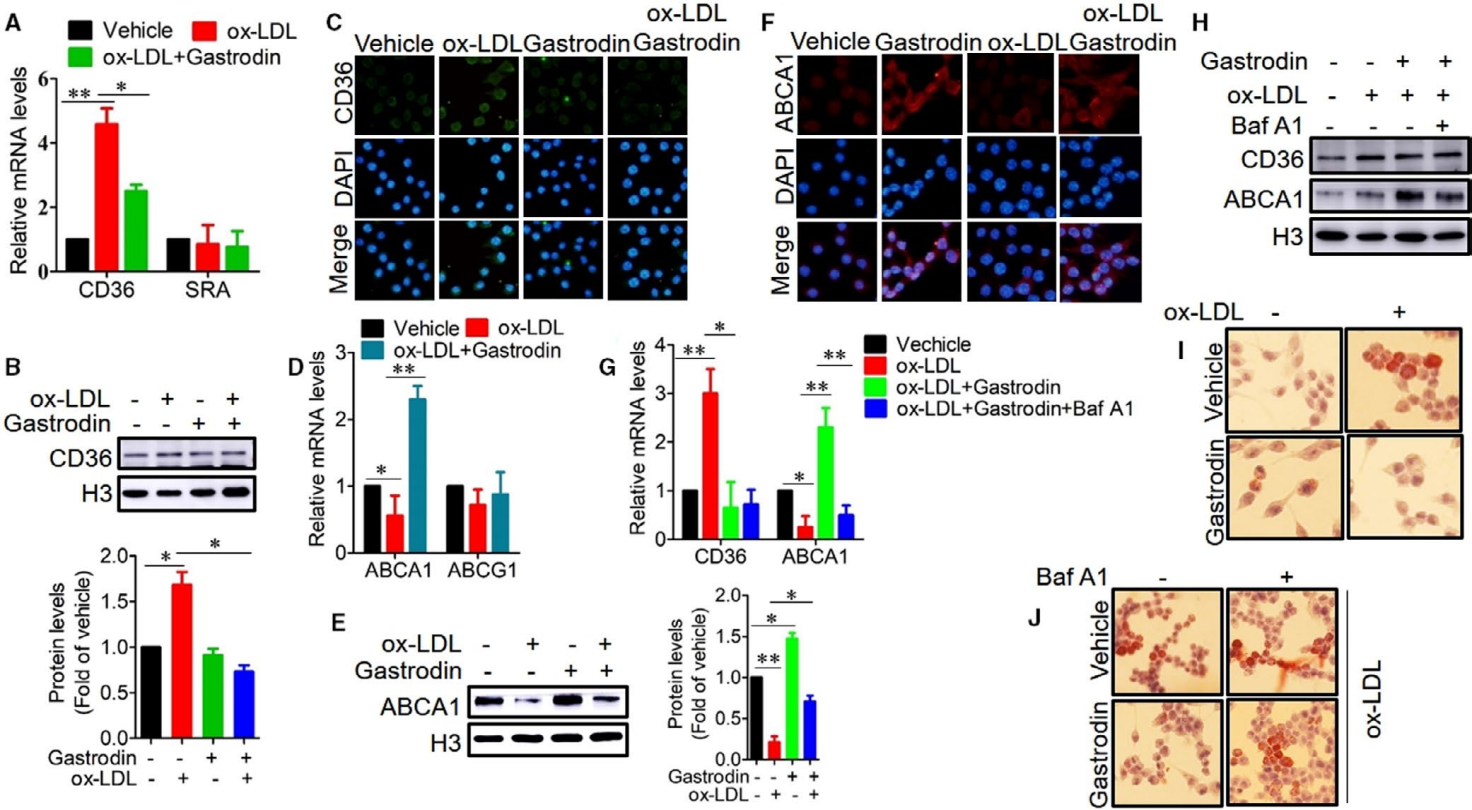
Gastrodin inhibits CD36‐dependent lipid uptake and promotes ABCA1‐dependent cholesterol efflux. A‐C, Gastrodin‐inhibited lipid uptake by down‐regulating the expression of CD36, but not SR‐A. The mRNA levels A, and protein levels B and C, were determined by RT‐PCR, Western blotting and immunofluorescence. D‐F, Gastrodin promoted cholesterol efflux through up‐regulating the expression of ABCA1, but not ABCG1. The mRNA levels D, and protein levels E and F, were determined by RT‐PCR, Western blotting and immunofluorescence. G and H, Macrophages were pre‐treated with Baf A1 (25nM) and then treated with gastrodin and ox‐LDL. The mRNA levels G, and protein levels H, of CD36 and ABCA1 were determined. I and J, Gastrodin inhibited the lipid accumulation and formation of foam cells through autophagy. Macrophages were treated with gastrodin and ox‐LDL in the presence or absence of Baf A1 and then stained with an Oil Red O stain kit. I, Gastrodin inhibited the lipid accumulation and formation of foam cells. J, The inhibition of autophagy aggravated the lipid accumulation and formation of foam cells in the presence of gastrodin. *P < .05; **P < .01. Results are means ± SD of three independent experiments. The value represents fold of vehicle

Macrophage cholesterol metabolism is closely linked to autophagy and lysosome function.^11^ As impaired cholesterol metabolism leads to intracellular lipid accumulation, promoting the formation of foam cells,^45^, ^46^ we have been suggested that gastrodin could affect macrophage cholesterol metabolism to regulate the formation of foam cells. Macrophage scavenger receptors (SRs), such as class A SR (SR‐A) and class B SR (CD36), are the primary receptors for the uptake and internalization of modified lipoproteins.^47^ We found that the mRNA and protein expression levels of CD36 were increased in the foam cells, but not SR‐A, and gastrodin prominently decreased the mRNA and protein expression levels of CD36 (Figure 3A and 3B). Similar to these results, the immunofluorescence of CD36 also showed an increased signal in the foam cells, which was reduced after gastrodin treatment (Figure 3C). Lysosome dysfunction leads to an impairment of cholesterol efflux from macrophages.^5^ The cholesterol transporters ATP‐binding cassette, sub‐family A, member 1 (ABCA1) and ATP‐binding cassette, sub‐family G, member 1 (ABCG1) are mainly responsible for transporting free cholesterol and phospholipids to apolipoprotein A‐I (ApoA‐I) and high‐density lipoprotein, respectively.^22^ We found that the mRNA and protein expression levels of ABCA1 were reduced in the foam cells, but not ABCG1, which were reversed after gastrodin treatment (Figure 3D and 3E). The immunofluorescence data of ABCA1 were also similar to the above (Figure 3F). These results demonstrate that gastrodin inhibits CD36‐dependent lipid uptake and promotes ABCA1‐dependent cholesterol efflux.

Next, we inhibited autophagy by Baf A1 and found that Baf A1 effectively reduced the mRNA and protein expression levels of ABCA1, but not affecting the expression of CD36 (Figure 3G and 3H), indicating that gastrodin promotes ABCA1‐dependent cholesterol efflux through autophagy. We then explored the effect of gastrodin on the lipid accumulation and formation of foam cells, observing the lowered lipid accumulation and the reduced formation of foam cells as demonstrated by ORO staining (Figure 3I). The inhibition of autophagy exacerbated the lipid accumulation and the formation of foam cells (Figure 3J). Collectively, our results suggest that gastrodin reduces lipid accumulation via inhibition of lipid uptake and promotion of cholesterol efflux in an autophagy‐dependent and lysosomal function‐dependent mechanism, therefore, inhibiting the formation of foam cells.

### 3.5 Gastrodin inhibits inflammation through autophagy in the foam cells

It has been reported that the deficiency of autophagy‐lysosomal system in foam cells contributes to a dramatic hyper‐inflammatory state, leading to advanced atherosclerosis.^12^, ^16^, ^23^ In contrast to control macrophages, the mRNA and protein expression levels of pro‐inflammatory cytokines, including IL‐1β and IL‐18, were increased in the foam cells, but not IL‐6 and TNF‐α. Gastrodin treatment significantly reduced the mRNA and protein expression levels of IL‐1β and IL‐18 (Figure S2A and S2B). Next, inhibiting autophagy by Baf A1, we observed that the mRNA and protein expression levels of IL‐1β and IL‐18 were prominently increased in the foam cells treated with gastrodin (Figure S2C and S2D). It is well known that cytokines and chemokines derived from foam cells can induce additional recruitment of circulating immune cells into subendothelial space, further increasing the formation of foam cells.^48^, ^49^ Therefore, our results suggest that autophagy is an important mechanism by which gastrodin inhibits inflammation to prevent the foam cell formation.

### 3.6 Gastrodin promotes TFEB activation in the foam cells

Next, we explored the mechanism by which gastrodin‐induced lysosome biogenesis and enhanced autophagic activity in the foam cells. Studies have shown that TFEB is involved in the regulation of lysosome biogenesis and autophagy, driving the expressions of autophagy‐related and lysosome‐related genes.^8^, ^50^ Normally, TFEB is phosphorylated and located in the cytosol in an inactive status, while dephosphorylated and translocated to the nucleus in response to environmental stimuli.^51^ In our study, we first examined the mRNA and total protein expression levels of TFEB. We found that their expressions were reduced in the foam cells, while gastrodin treatment rescued their expressions (Figure S3A and S3B). Next, we examined the subcellular localization of TFEB. We observed that the protein level of TFEB was reduced in the nucleus of foam cells, while gastrodin treatment increased TFEB protein level (Figure S3C). Immunofluorescence analysis further demonstrated that TFEB was mainly located in the cytoplasm of foam cells, and gastrodin treatment promoted nuclear translocation of TFEB (Figure S3D). Taken together, our results suggest that gastrodin promotes TFEB activation in the foam cells.

### 3.7 TFEB is involved in the effect of gastrodin on lysosome biogenesis and autophagy

**FIGURE 4:**
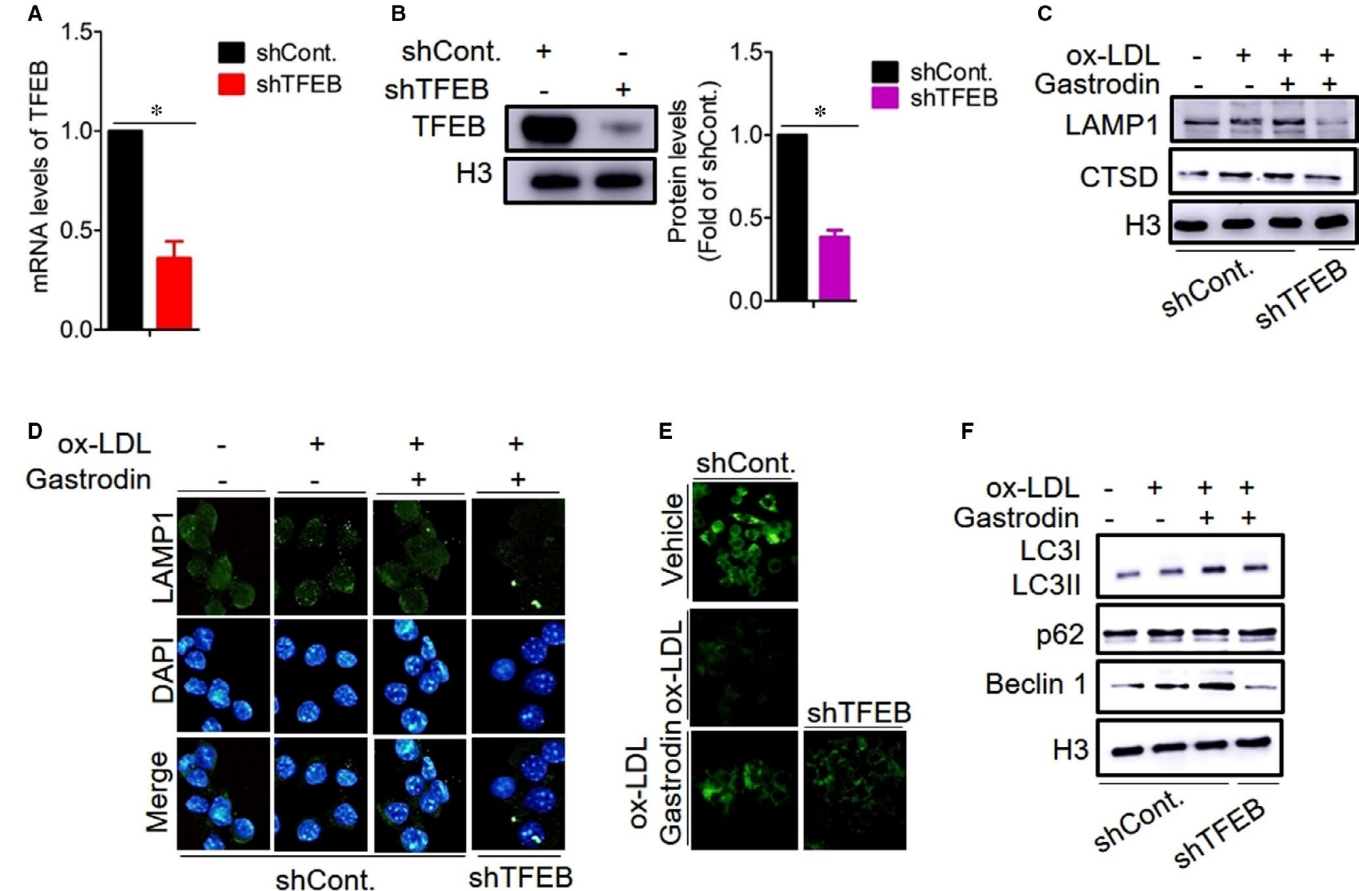
TFEB is involved in gastrodin‐induced lysosome biogenesis and autophagy. A and B, The expression of TFEB was determined after transfecting by control shRNA and shRNA against TFEB. A, The mRNA level of TFEB. B, The protein level of TFEB. C‐E, Inhibition of TFEB expression abolished the effect of gastrodin on lysosomal biogenesis and lysosomal function in the foam cells. C, The protein levels of LAMP1 and CTSD. D, Immunofluorescence analysis of LAMP1. E, Measurement of lysosomal pH. F, Inhibiting TFEB expression reduced the protein levels of LC3II and Beclin 1 and increased the protein level of p62. *P < .05. Results are presented as mean ± SD of three independent experiments. The value represents fold of vehicle. shCont., Control shRNA

Next, we knocked down TFEB by shRNA against TFEB, observing that the mRNA and protein expression levels of TFEB were significantly reduced (Figure 4A and 4B). By Western blotting and immunofluorescence analysis, we found that the knockdown of TFEB significantly inhibited LAMP1 protein level compared with the foam cells subjected to control shRNA in the presence of gastrodin (Figure 4C and 4D). Consistent with these results, the knockdown of TFEB also reversed the effect of gastrodin on CTSD protein expression and pH in the foam cells (Figure 4C and 4E), suggesting that gastrodin induces lysosomal biogenesis through TFEB to rescue lysosomal dysfunction. We then tested the expression levels of autophagy‐related proteins and observed that knockdown of TFEB resulted in decreased protein levels of LC3II and Beclin1 and increased p62 protein level (Figure 4F), suggesting that TFEB is involved in gastrodin‐induced autophagy in the foam cells. Collectively, these results demonstrate that gastrodin induces lysosome biogenesis and enhances autophagy activity in a TFEB‐dependent manner in the foam cells.

### 3.8 Gastrodin up‐regulates TFEB expression by increasing the phosphorylation and nuclear translocation of FoxO1

**FIGURE 5:**
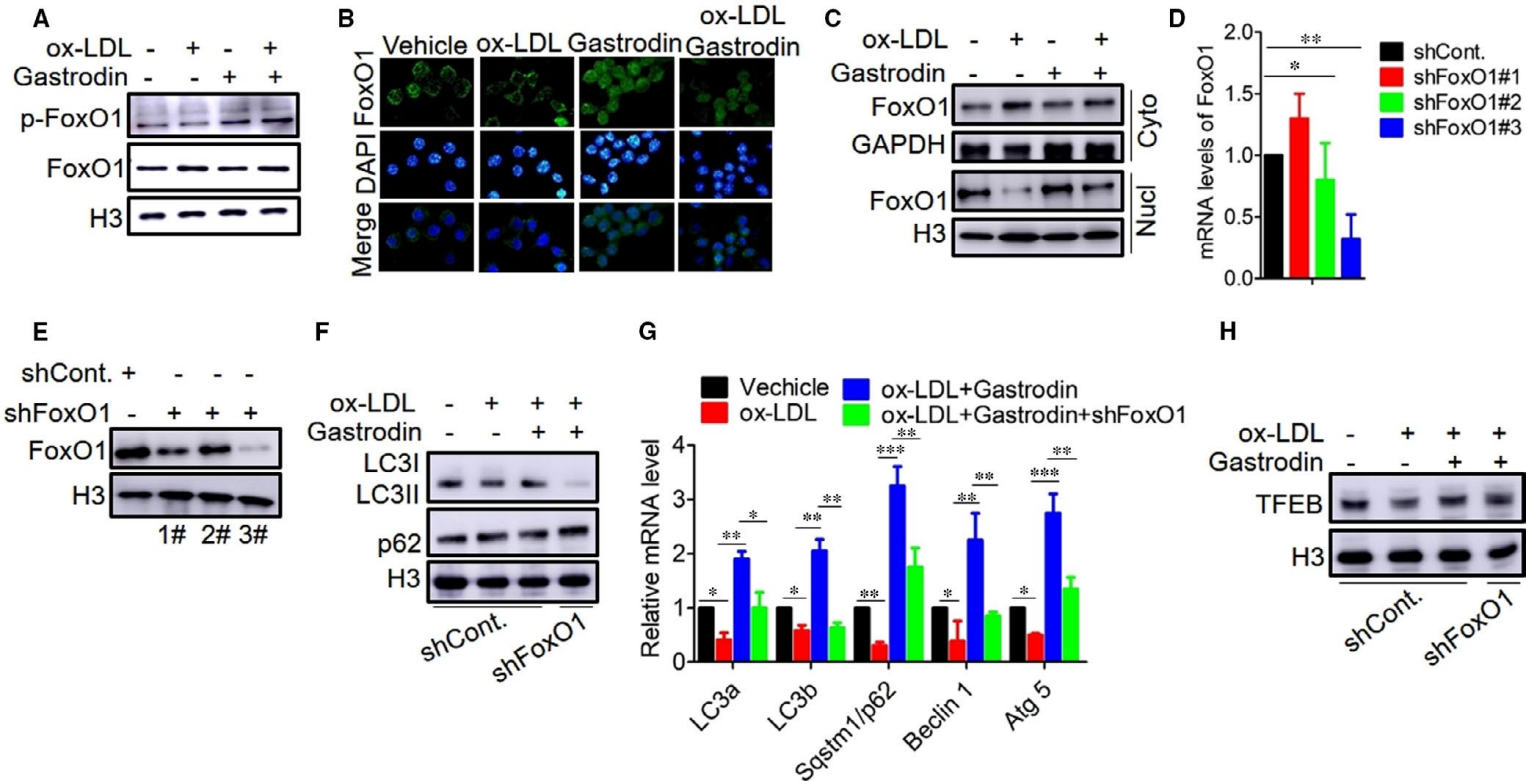
Gastrodin increases the phosphorylation and nuclear translocation of FoxO1 to up‐regulate TFEB expression in the foam cells. A, Gastrodin increased the phosphorylation of FoxO1 in the foam cells. B and C, Gastrodin promoted nuclear translocation of FoxO1 in the foam cells. B, Images of the subcellular locations of FoxO1. C, Representative blots of cytoplasmic and nuclear fractions of FoxO1. D and E, The determination of mRNA and protein expression levels of FoxO1 after transfecting by control shRNA and shFoxO1. D, Measurement of mRNA expression level by RT‐PCR. E, Measurement of protein expression level by Western blotting. F and G, Inhibition of FoxO1 expression abolished the effect of gastrodin on autophagy in the foam cells. The protein levels of LC3I/II and p62 F, and mRNA levels of autophagic genes G, were determined by Western blotting and RT‐PCR. H, Inhibition of FoxO1 reduced the protein level of TFEB. *P < .05; **P < .01; ***P < .001. Results are presented as mean ±SD of three independent experiments. The value represents fold of vehicle. shCont., Control shRNA. Cyto, Cytoplasm; Nucl, Nucleus

Several studies have reported that FoxO (forkhead box class O) family members can induce autophagy via binding to the promoter regions of autophagy genes and transactivating their expressions.^36^, ^52^, ^53^ We found that the phosphorylation level of FoxO1 was reduced in the foam cells compared to control macrophages, and gastrodin treatment significantly increased the phosphorylation level of FoxO1 (Figure 5A). Given the fact that FoxO‐induced autophagy gene expression requires FoxO translocation from the cytoplasm into the nucleus,^54^ we thus determined the subcellular distribution of FoxO1. As shown in Figure 5B and 5C, gastrodin treatment promoted FoxO1 nuclear retention, indicating that gastrodin induces FoxO1 activation in the foam cells. Next, we knocked down FoxO1 by shRNA and observed that the mRNA and protein expression levels of FoxO1 were markedly inhibited in the macrophages subjected to shFoxO1 3# (Figure 5D and 5E). Meanwhile, we found that inhibition of FoxO1 expression resulted in the reduced LC3II level and increased p62 level in the foam cells treated with gastrodin (Figure 5F). Consistent with these results, we also found that the mRNA expression levels of autophagy genes were markedly reduced after inhibiting the expression of FoxO1 (Figure 5G), suggesting that FoxO1 is involved in gastrodin‐induced autophagy. Next, to validate the relationship between FoxO1 and TFEB, we determined the protein expression of TFEB in the foam cells subjected to shFoxO1 in the presence of gastrodin, observing a reduced protein level of TFEB (Figure 5H). These results suggest that gastrodin up‐regulates TFEB expression by inducing the phosphorylation and nuclear translocation of FoxO1.

### 3.9 AMPK acts as the upstream molecular regulator of FoxO1 and TFEB

**FIGURE 6:**
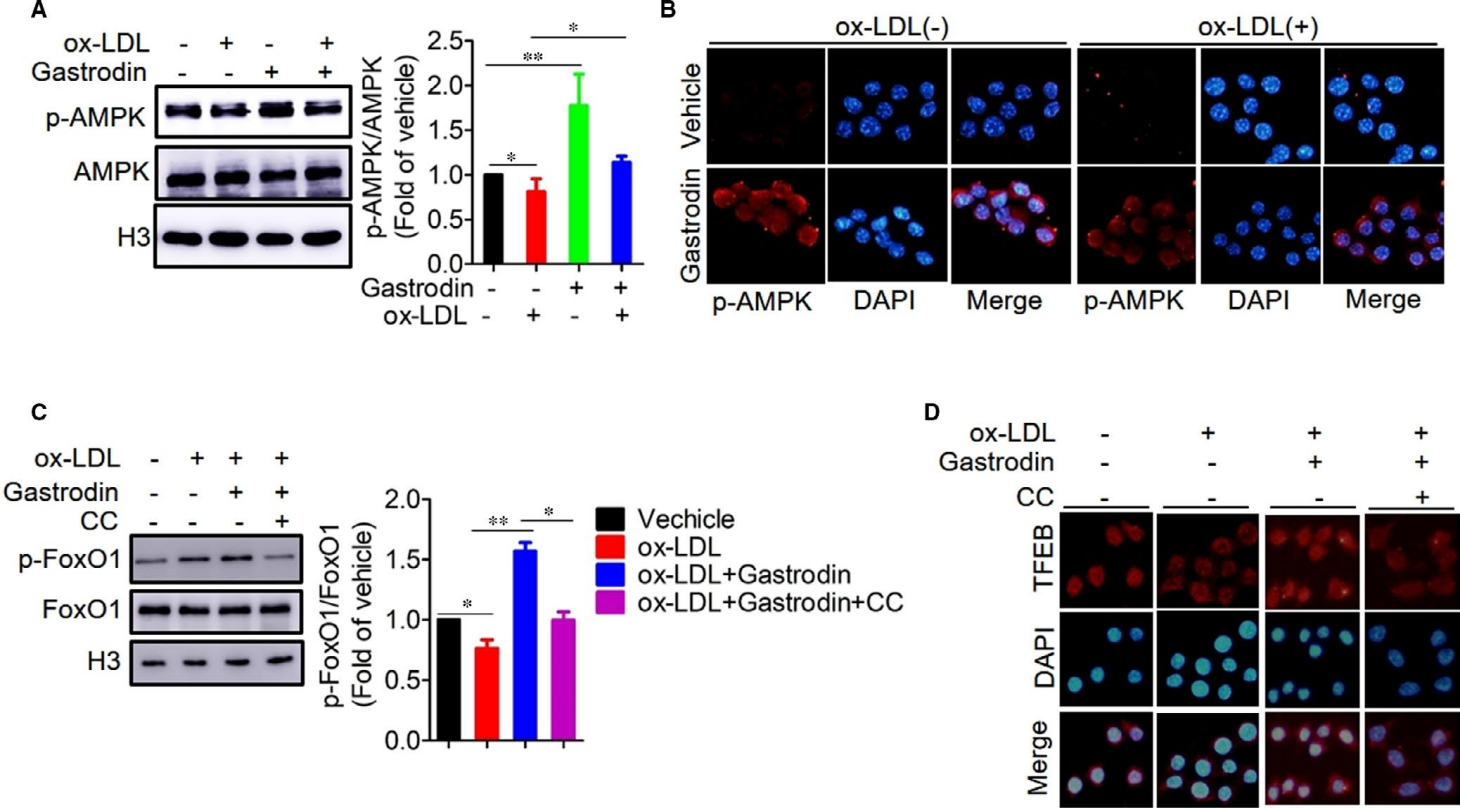
AMPK is a critical upstream regulator of FoxO1 and TFEB. A and B, Gastrodin activated AMPK in the foam cells. A, Representative blots of AMPK and p‐AMPK in macrophages. B, Immunofluorescence analysis of p‐AMPK in macrophages. C and D, The inhibition of AMPK activity decreased the phosphorylation of FoxO1 and nuclear translocation of TFEB. Macrophages were treated with CC (10μM) for 1 h. The phosphorylation level of FoxO1 was analysed by Western blotting C, and nuclear translocation of TFEB was determined by immunofluorescence D. *P < .05; **P < .01. Results are presented as mean ± SD of three independent experiments. The value represents fold of vehicle. CC, Dorsomorphin dihydrochloride

Previous studies have demonstrated that AMPK can regulate autophagy.^55^, ^56^ We thus investigated whether gastrodin‐regulated lysosomal biogenesis and autophagy required AMPK. In the foam cells, the phosphorylation level of AMPK was decreased as compared with control macrophages, while gastrodin treatment significantly increased the phosphorylation level of AMPK (Figure 6A). Consistent with these results, immunofluorescence analysis also demonstrated a decreased phosphorylation level of AMPK in the foam cells in the absence of gastrodin and an elevated phosphorylation level in the presence of gastrodin (Figure 6B). Next, we inhibited AMPK activity by using Dorsomorphin dihydrochloride (CC), a pharmacological inhibitor of AMPK activity, and found that gastrodin failed to increase FoxO1 phosphorylation level in the foam cells (Figure 6C). At the same time, we also found that the inhibition of AMPK activity prevented the nuclear translocation of TFEB in the foam cells treated with gastrodin (Figure 6D). Taken together, these results demonstrate that AMPK functions as an upstream molecular regulator of FoxO1 and TFEB.

## 4 DISCUSSION

**FIGURE 7:**
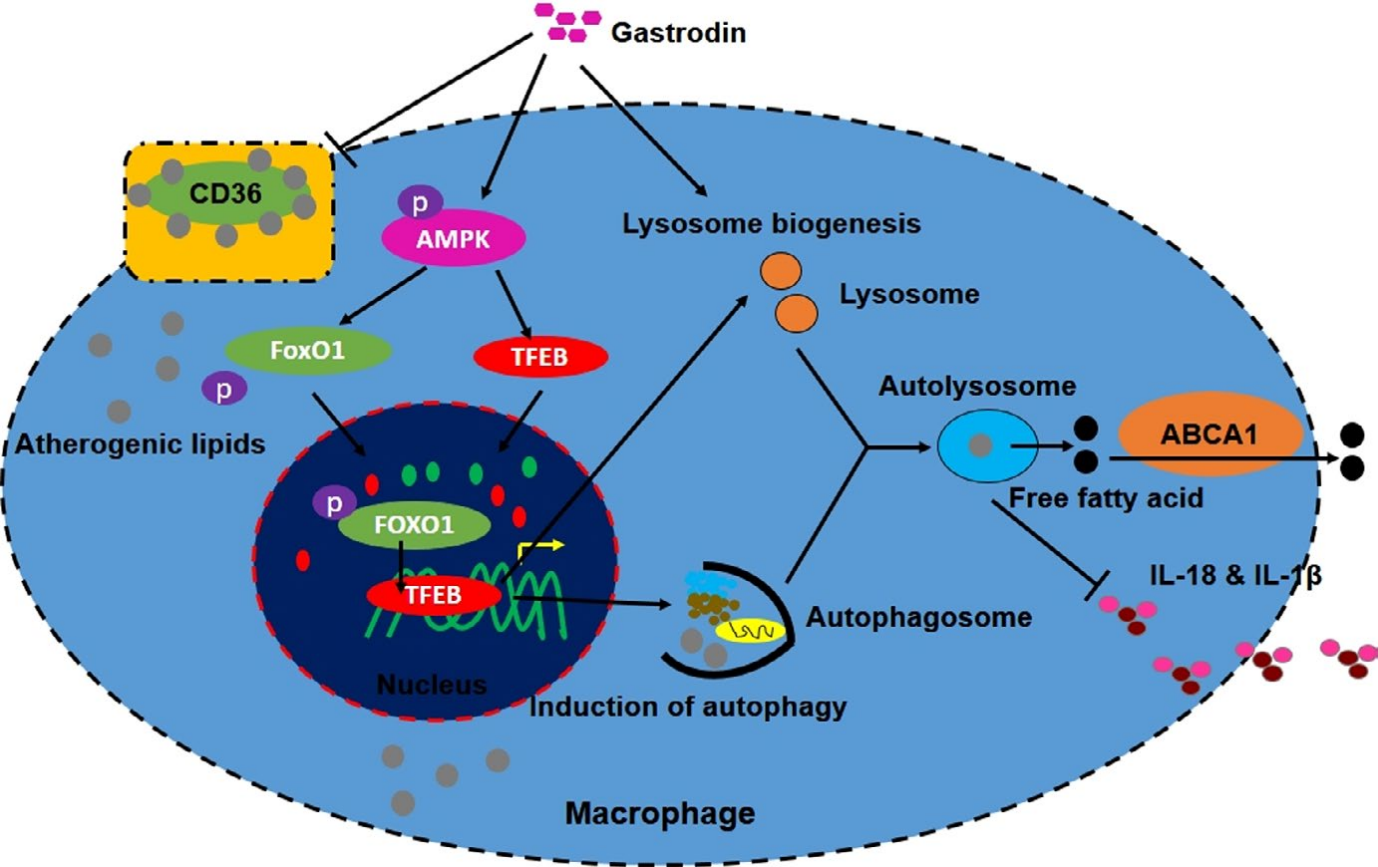
Schematic model for gastrodin‐inhibited formation of foam cells through inducing lysosomal biogenesis and autophagy via AMPK‐FoxO1‐TFEB signalling axis

In this study, we characterized for the first time a novel mechanism by which gastrodin inhibits the formation of foam cells (Figure 7). In an in vitro foam cell model, we demonstrated that gastrodin induces lysosomal biogenesis and autophagy through TFEB to rescue the lysosomal dysfunction and autophagy deficiency of foam cells. Restoration of lysosome function and autophagic activity both decrease intracellular lipid content and inflammation to inhibit the formation of foam cells. Mechanistically, gastrodin activates AMPK, increasing the phosphorylation and nuclear translocation of FoxO1, and the latter further increases TFEB expression.

In macrophages, ox‐LDL is taken up by several scavenger receptors (SRs) and transported into endosomes‐lysosomes. In the endosomes‐lysosomes, cholesteryl ester is converted to free cholesterol (FC) by lysosomal acid lipase.^22^ But excessive FC in the lysosomes can result in loss of lysosomal acidification.^5^ Furthermore, the accumulation of ox‐LDL in macrophages can result in inactivation of the lysosomal proteases, such as cathepsins (cathepsin B and D), impairing lysosomal degradation capacity.^5^, ^57^ Recently, two studies have shown that induction of lysosomal biogenesis by genetic or pharmacological intervention can rescue lysosomal dysfunction in atherosclerotic macrophages.^5^, ^9^ In this study, our results showed decreased expression of CTSD and increased pH in the foam cells, indicating lysosomal dysfunction. Gastrodin‐induced lysosomal biogenesis to rescue atherogenic lipid‐induced lysosomal dysfunction. The restoration of lysosomal function in macrophages promotes cholesterol efflux and reduces the production of pro‐inflammatory cytokines,^5^ therefore, decreasing lipid accumulation and preventing the formation of foam cells. Our results demonstrated that inducing lysosomal biogenesis to restore lysosomal function is one of the mechanisms by which gastrodin inhibits the formation of foam cells.

Autophagy serves as an essential cellular adaptation mechanism.^10^ Accumulation of atherogenic lipids results in impaired autophagy in plaque macrophages, followed by dramatic progression of atherosclerosis.^16^ In our study, we found the impaired autophagy in the foam cells, while gastrodin significantly restored autophagy activity of foam cells. Inhibiting autophagy can prevent cholesterol efflux and induce selective secretion of IL‐1β, but not TNF‐α, in foam cells.^16^ Our data demonstrated that gastrodin up‐regulated ABCA1 expression to increase cholesterol efflux and reduced pro‐inflammatory cytokines IL‐1β and IL‐18 secretion, but not IL‐6 and TNF‐α, in an autophagy‐dependent manner. Thus, our results suggested that enhanced autophagy activity is a critical mechanism by which gastrodin inhibits the formation of foam cells.

TFEB is a known master regulator of autophagy and lysosomal biogenesis.^5^ During atherosclerosis progression, lipid overload in macrophages for prolonged periods leads to TFEB desensitization.^5^ Our results showed decreased TFEB expression, including mRNA and protein levels, in the foam cells. Simultaneously, our results also showed decreased nuclear translocation of TFEB in the foam cells, indicating that TFEB is inactivated in the foam cells. Previous studies have demonstrated that macrophage‐specific TFEB overexpression can restore dysfunctional lysosomes and impaired autophagy.^5^, ^9^ In our results, we showed that gastrodin promoted TFEB activation and increased TFEB expression to rescue lysosomal dysfunction and autophagy deficiency, while the knockdown of TFEB abolished the effect of gastrodin, demonstrating that TFEB signalling is essential to gastrodin‐induced lysosomal biogenesis and autophagy. Furthermore, our results showed that gastrodin decreased lipid accumulation via inhibited lipid uptake and increased cholesterol efflux. However, inhibiting autophagy did not affect lipid uptake and only affected cholesterol efflux, suggesting that gastrodin may regulate lipid uptake through other mechanisms beside autophagy, necessitating further investigation in the future. Taken together, our data demonstrated that TFEB is critical for gastrodin inhibition of foam cell formation.

Although TFEB activation by genetic or pharmacological methods can alleviate atherosclerosis,^5^, ^9^, ^11^ the molecular basis for regulation of TFEB activation is unknown. Recently, two studies have reported that FoxO induces autophagy through up‐regulated expression of autophagy genes in chondrocytes and cardiomyocytes.^53^, ^58^ Moreover, FoxO‐induced expression of autophagy genes requires nuclear translocation of FoxO from the cytosolic compartment.^54^ Our results showed that gastrodin increased the phosphorylation level of FoxO1 and promoted its nuclear translocation, while the knockdown of FoxO1 inhibited the expression of autophagy genes, indicating that FoxO1 is involved in gastrodin‐induced autophagy. Notably, AMPK can directly phosphorylate FoxO1 and promotes its nuclear translocation, increasing downstream target gene expression.^59^ A subsequent study has demonstrated that FoxO1 regulates TFEB expression via direct binding to three insulin response elements of TFEB promoter region.^60^ Our results demonstrated that gastrodin increased the phosphorylation of FoxO1 and promoted its nuclear translocation by activating AMPK and that the knockdown of FoxO1 reduced TFEB expression. Thus, our data demonstrated that gastrodin increases TFEB expression via AMPK‐FoxO1 signalling.

In conclusion, our findings demonstrated that gastrodin exerts an atheroprotective role via a novel mechanism: gastrodin induces lysosomal biogenesis and enhances autophagic activity through an AMPK‐FoxO1‐TFEB signal axis to inhibit foam cell formation. Although our study is based on in vitro data, these findings provide mechanistic insights into gastrodin's positive effect on foam cell formation and establish the validity of gastrodin as a potential treatment of atherosclerotic vascular disease. Furthermore, the effect of gastrodin on atherosclerotic mice is now under investigation in our laboratory and is expected to validate our in vitro findings and further confirm gastrodin's therapeutic potential.

## CONFLICT OF INTEREST

The authors declare that they have no competing interests.

## AUTHOR CONTRIBUTIONS

**Jun Tao:** Data curation (equal); Formal analysis (equal); Investigation (equal); Methodology (equal); Visualization (equal); Writing‐original draft (equal). **Ping Yang:** Data curation (equal); Formal analysis (equal); Investigation (equal); Methodology (equal); Writing‐original draft (equal). **Liqiu Xie:** Formal analysis (equal); Investigation (equal); Writing‐original draft (equal). **Yuwei Pu:** Formal analysis (equal); Investigation (equal); Writing‐original draft (equal). **Jiazhi Guo:** Formal analysis (equal); Investigation (equal). **Jianlin Jiao:** Formal analysis (equal); Investigation (equal). **Lin Sun:** Conceptualization (equal); Funding acquisition (equal); Project administration (equal); Resources (equal); Writing‐original draft (equal). **Di Lu:** Conceptualization (equal); Data curation (equal); Formal analysis (equal); Funding acquisition (equal); Project administration (equal); Resources (equal); Writing‐original draft (equal).

## Supporting information

Fig S1

Fig S2

Fig S3

Table S1‐S2

Supplementary Material

Supplementary Material

## Data Availability

All data generated or analysed during this study are included in this article.

## References

[1] Zhou J , Tan S‐H , Nicolas V , et al. Activation of lysosomal function in the course of autophagy via mTORC1 suppression and autophagosome‐lysosome fusion. Cell Res. 2013;23(4):508‐523. 23337583 10.1038/cr.2013.11 PMC3616426

[2] Sardiello M , Palmieri M , di Ronza A , et al. A gene network regulating lysosomal biogenesis and function. Science. 2009;325(5939):473‐477. 19556463 10.1126/science.1174447

[3] Wang W , Gao Q , Yang M , et al. Up‐regulation of lysosomal TRPML1 channels is essential for lysosomal adaptation to nutrient starvation. Proc Natl Acad Sci USA. 2015;112(11):E1373‐E1381. 25733853 10.1073/pnas.1419669112 PMC4371935

[4] Leeman DS , Hebestreit K , Ruetz T , et al. Lysosome activation clears aggregates and enhances quiescent neural stem cell activation during aging. Science. 2018;359(6381):1277‐1283. 29590078 10.1126/science.aag3048 PMC5915358

[5] Emanuel R , Sergin I , Bhattacharya S , et al. Induction of lysosomal biogenesis in atherosclerotic macrophages can rescue lipid‐induced lysosomal dysfunction and downstream sequelae. Arterioscler Thromb Vasc Biol. 2014;34(9):1942‐1952. 25060788 10.1161/ATVBAHA.114.303342 PMC4140993

[6] Platt FM , Andria G , Ballabio A . Lysosomal storage diseases. Nat Rev Dis Primers. 2018;4(1):27. 30275469 10.1038/s41572-018-0025-4

[7] Kao AW , McKay A , Singh PP , Brunet A , Huang EJ . Progranulin, lysosomal regulation and neurodegenerative disease. Nat Rev Neurosci. 2017;18(6):325‐333. 28435163 10.1038/nrn.2017.36 PMC6040832

[8] Martini‐Stoica H , Xu Y , Ballabio A , Zheng H . The Autophagy‐Lysosomal Pathway in Neurodegeneration: A TFEB Perspective. Trends Neurosci. 2016;39(4):221‐234. 26968346 10.1016/j.tins.2016.02.002 PMC4928589

[9] Sergin I , Evans TD , Zhang X , et al. Exploiting macrophage autophagy‐lysosomal biogenesis as a therapy for atherosclerosis. Nat Commun. 2017;8:15750. 28589926 10.1038/ncomms15750 PMC5467270

[10] Lee JM , Wagner M , Xiao R , et al. Nutrient‐sensing nuclear receptors coordinate autophagy. Nature. 2014;516(7529):112‐115. 25383539 10.1038/nature13961 PMC4267857

[11] Li X , Zhang X , Zheng L , et al. Hypericin‐mediated sonodynamic therapy induces autophagy and decreases lipids in THP‐1 macrophage by promoting ROS‐dependent nuclear translocation of TFEB. Cell Death Dis. 2016;7(12):e2527. 28005078 10.1038/cddis.2016.433 PMC5260986

[12] Ouimet M , Franklin V , Mak E , Liao X , Tabas I , Marcel YL . Autophagy regulates cholesterol efflux from macrophage foam cells via lysosomal acid lipase. Cell Metab. 2011;13(6):655‐667. 21641547 10.1016/j.cmet.2011.03.023 PMC3257518

[13] Dubland JA , Francis GA . Lysosomal acid lipase: at the crossroads of normal and atherogenic cholesterol metabolism. Front Cell Dev Biol. 2015;3:3. 25699256 10.3389/fcell.2015.00003 PMC4313778

[14] Visvikis O , Ihuegbu N , Labed SA , et al. Innate host defense requires TFEB‐mediated transcription of cytoprotective and antimicrobial genes. Immunity. 2014;40(6):896‐909. 24882217 10.1016/j.immuni.2014.05.002 PMC4104614

[15] Luo R , Su LY , Li G , et al. Activation of PPARA‐mediated autophagy reduces Alzheimer disease‐like pathology and cognitive decline in a murine model. Autophagy. 2020;16(1):52‐69. 30898012 10.1080/15548627.2019.1596488 PMC6984507

[16] Razani B , Feng C , Coleman T , et al. Autophagy links inflammasomes to atherosclerotic progression. Cell Metab. 2012;15(4):534‐544. 22440612 10.1016/j.cmet.2012.02.011 PMC3322320

[17] Wang L , Jiang Y , Song X , et al. Pdcd4 deficiency enhances macrophage lipoautophagy and attenuates foam cell formation and atherosclerosis in mice. Cell Death Dis. 2016;7:e2055. 26775706 10.1038/cddis.2015.416 PMC4816189

[18] Lu H , Fan Y , Qiao C , et al. TFEB inhibits endothelial cell inflammation and reduces atherosclerosis. Sci Signal. 2017;10(464). 10.1126/scisignal.aah4214 28143903

[19] Huang L , Chambliss KL , Gao X , et al. SR‐B1 drives endothelial cell LDL transcytosis via DOCK4 to promote atherosclerosis. Nature. 2019;569(7757):565‐569. 31019307 10.1038/s41586-019-1140-4 PMC6631346

[20] Wang J , Ma A , Zhao M , Zhu H . AMPK activation reduces the number of atheromata macrophages in ApoE deficient mice. Atherosclerosis. 2017;258:97‐107. 28235712 10.1016/j.atherosclerosis.2017.01.036

[21] Lusis AJ . Atherosclerosis. Nature. 2000;407(6801):233‐241. 11001066 10.1038/35025203 PMC2826222

[22] Tabas I , Bornfeldt KE . Macrophage Phenotype and Function in Different Stages of Atherosclerosis. Circ Res. 2016;118(4):653‐667. 26892964 10.1161/CIRCRESAHA.115.306256 PMC4762068

[23] Liao X , Sluimer JC , Wang Y , et al. Macrophage autophagy plays a protective role in advanced atherosclerosis. Cell Metab. 2012;15(4):545‐553. 22445600 10.1016/j.cmet.2012.01.022 PMC3322248

[24] Bobryshev YV , Shchelkunova TA , Morozov IA , et al. Changes of lysosomes in the earliest stages of the development of atherosclerosis. J Cell Mol Med. 2013;17(5):626‐635. 23490339 10.1111/jcmm.12042 PMC3822815

[25] Li W , Yuan XM , Olsson AG , Brunk UT , et al. Uptake of oxidized LDL by macrophages results in partial lysosomal enzyme inactivation and relocation. Arterioscler Thromb Vasc Biol. 1998;18(2):177‐184. 9484981 10.1161/01.atv.18.2.177

[26] Ma M , Song L , Yan H , et al. Low dose tunicamycin enhances atherosclerotic plaque stability by inducing autophagy. Biochem Pharmacol. 2016;100:51‐60. 26616221 10.1016/j.bcp.2015.11.020

[27] Shapiro MD , Fazio S . From lipids to inflammation: new approaches to reducing atherosclerotic risk. Circ Res. 2016;118(4):732‐749. 26892970 10.1161/CIRCRESAHA.115.306471

[28] Chen J , Huang Y , Hu X , Bian X , Nian S . Gastrodin prevents homocysteine‐induced human umbilical vein endothelial cells injury via PI3K/Akt/eNOS and Nrf2/ARE pathway. J Cell Mol Med. 2020. 10.1111/jcmm.16073 PMC7810955 33320446

[29] Yang P , Han Y , Gui L , et al. Gastrodin attenuation of the inflammatory response in H9c2 cardiomyocytes involves inhibition of NF‐kappaB and MAPKs activation via the phosphatidylinositol 3‐kinase signaling. Biochem Pharmacol. 2013;85(8):1124‐1133. 23376120 10.1016/j.bcp.2013.01.020

[30] Qu LL , Yu B , Li Z , Jiang W‐X , Jiang J‐D , Kong W‐J . Gastrodin ameliorates oxidative stress and proinflammatory response in nonalcoholic fatty liver disease through the AMPK/Nrf2 pathway. Phytother Res. 2016;30(3):402‐411. 26634892 10.1002/ptr.5541

[31] Wang Y , Wu Z , Liu X , Fu Q . Gastrodin ameliorates Parkinson's disease by downregulating connexin 43. Mol Med Rep. 2013;8(2):585‐590. 23783886 10.3892/mmr.2013.1535

[32] Hu Y , Li C , Shen W . Gastrodin alleviates memory deficits and reduces neuropathology in a mouse model of Alzheimer's disease. Neuropathology. 2014;34(4):370‐377. 24661139 10.1111/neup.12115

[33] Geng, Y. N. , Bin, Y. U. , Zuo, Z. , & Kong, W. Experimental study of Gastrodia Powder in improving hepatic steatosis in rats intragastrically administered with fat emulsion. China Medical Herald. 2013;10(30):11–15.

[34] Geng, Y. , Bin, Y. U. , & Kong, W. Gastrodin ameliorates oleic acid‐induced fat accumulation through activation of AMPK pathway in HL‐7702 cells. Chinese Pharmacological Bulletin. 2015;31(1):39‐44.

[35] Zhang J , Wang J , Wong YK , et al. Docetaxel enhances lysosomal function through TFEB activation. Cell Death Dis. 2018;9(6):614. 29795139 10.1038/s41419-018-0571-4 PMC5966422

[36] Wang S , Xia P , Huang G , et al. FoxO1‐mediated autophagy is required for NK cell development and innate immunity. Nat Commun. 2016;7:11023. 27010363 10.1038/ncomms11023 PMC4820827

[37] Li AC , Binder CJ , Gutierrez A , et al. Differential inhibition of macrophage foam‐cell formation and atherosclerosis in mice by PPARalpha, beta/delta, and gamma. J Clin Invest. 2004;114(11):1564‐1576. 15578089 10.1172/JCI18730 PMC529277

[38] Qi X , Man SM , Malireddi RKS , et al. Cathepsin B modulates lysosomal biogenesis and host defense against Francisella novicida infection. J Exp Med. 2016;213(10):2081‐2097. 27551156 10.1084/jem.20151938 PMC5030800

[39] Zhou C , Zhong W , Zhou J , et al. Monitoring autophagic flux by an improved tandem fluorescent‐tagged LC3 (mTagRFP‐mWasabi‐LC3) reveals that high‐dose rapamycin impairs autophagic flux in cancer cells. Autophagy. 2012;8(8):1215‐1226. 22647982 10.4161/auto.20284

[40] Bae EJ , Yang NY , Lee C , Kim S , Lee HJ , Lee SJ . Haploinsufficiency of cathepsin D leads to lysosomal dysfunction and promotes cell‐to‐cell transmission of alpha‐synuclein aggregates. Cell Death Dis. 2015;6:e1901. 26448324 10.1038/cddis.2015.283 PMC4632307

[41] Li X , Zhu Q , Liu Y , Yang Z , Li B . Gastrodin protects myocardial cells against hypoxia/reoxygenation injury in neonatal rats by inhibiting cell autophagy through the activation of mTOR signals in PI3K‐Akt pathway. J Pharm Pharmacol. 2018;70(2):259‐267. 29148068 10.1111/jphp.12838

[42] Hipolito VEB , Diaz JA , Tandoc KV , et al. Enhanced translation expands the endo‐lysosome size and promotes antigen presentation during phagocyte activation. PLoS Biol. 2019;17(12):e3000535. 31800587 10.1371/journal.pbio.3000535 PMC6913987

[43] Ruck A , Attonito J , Garces KT , et al. The Atg6/Vps30/Beclin 1 ortholog BEC‐1 mediates endocytic retrograde transport in addition to autophagy in C. elegans. Autophagy. 2011;7(4):386‐400. 21183797 10.4161/auto.7.4.14391 PMC3108013

[44] Zhang J , Ng S , Wang J , et al. Histone deacetylase inhibitors induce autophagy through FOXO1‐dependent pathways. Autophagy. 2015;11(4):629‐642. 25919885 10.1080/15548627.2015.1023981 PMC4502718

[45] Brownell N , Rohatgi A . Modulating cholesterol efflux capacity to improve cardiovascular disease. Curr Opin Lipidol. 2016;27(4):398‐407. 27213627 10.1097/MOL.0000000000000317

[46] Oh J , Riek AE , Darwech I , et al. Deletion of macrophage Vitamin D receptor promotes insulin resistance and monocyte cholesterol transport to accelerate atherosclerosis in mice. Cell Rep. 2015;10(11):1872‐1886. 25801026 10.1016/j.celrep.2015.02.043 PMC4495012

[47] Manning‐Tobin JJ , Moore KJ , Seimon TA , et al. Loss of SR‐A and CD36 activity reduces atherosclerotic lesion complexity without abrogating foam cell formation in hyperlipidemic mice. Arterioscler Thromb Vasc Biol. 2009;29(1):19‐26. 18948635 10.1161/ATVBAHA.108.176644 PMC2666043

[48] Raggi P , Genest J , Giles JT , et al. Role of inflammation in the pathogenesis of atherosclerosis and therapeutic interventions. Atherosclerosis. 2018;276:98‐108. 30055326 10.1016/j.atherosclerosis.2018.07.014

[49] Mallat Z , Corbaz A , Scoazec A , et al. Interleukin‐18/interleukin‐18 binding protein signaling modulates atherosclerotic lesion development and stability. Circ Res. 2001;89(7):E41‐E45. 11577031 10.1161/hh1901.098735

[50] Kim SH , Kim G , Han DH , et al. Ezetimibe ameliorates steatohepatitis via AMP activated protein kinase‐TFEB‐mediated activation of autophagy and NLRP3 inflammasome inhibition. Autophagy. 2017;13(10):1767‐1781. 28933629 10.1080/15548627.2017.1356977 PMC5640190

[51] Settembre C , Di Malta C , Polito VA , et al. TFEB links autophagy to lysosomal biogenesis. Science. 2011;332(6036):1429‐1433. 21617040 10.1126/science.1204592 PMC3638014

[52] Cheng Z . The FoxO–Autophagy Axis in Health and Disease. Trends Endocrinol Metab. 2019;30(9):658‐671. 31443842 10.1016/j.tem.2019.07.009

[53] Matsuzaki T , Alvarez‐Garcia O , Mokuda S , et al. FoxO transcription factors modulate autophagy and proteoglycan 4 in cartilage homeostasis and osteoarthritis. Sci Transl Med. 2018;10(428):eaan0746. 29444976 10.1126/scitranslmed.aan0746 PMC6204214

[54] Cheng Z . The FoxO‐Autophagy Axis in Health and Disease. Trends Endocrinol Metab. 2019;30(9):658‐671. 31443842 10.1016/j.tem.2019.07.009

[55] Fu S , Chen L , Wu Y , et al. Gastrodin pretreatment alleviates myocardial ischemia/reperfusion injury through promoting autophagic flux. Biochem Biophys Res Commun. 2018;503(4):2421‐2428. 29969626 10.1016/j.bbrc.2018.06.171

[56] Kim J , Kundu M , Viollet B , Guan K‐L . AMPK and mTOR regulate autophagy through direct phosphorylation of Ulk1. Nat Cell Biol. 2011;13(2):132‐141. 21258367 10.1038/ncb2152 PMC3987946

[57] Hoppe G , O'Neil J , Hoff HF . Inactivation of lysosomal proteases by oxidized low density lipoprotein is partially responsible for its poor degradation by mouse peritoneal macrophages. J Clin Invest. 1994;94(4):1506‐1512. 7929826 10.1172/JCI117490 PMC295294

[58] Ning Y , Li Z , Qiu Z . FOXO1 silence aggravates oxidative stress‐promoted apoptosis in cardiomyocytes by reducing autophagy. J Toxicol Sci. 2015;40(5):637‐645. 26354380 10.2131/jts.40.637

[59] Yun H , Park S , Kim M‐J , et al. AMP‐activated protein kinase mediates the antioxidant effects of resveratrol through regulation of the transcription factor FoxO1. FEBS J. 2014;281(19):4421‐4438. 25065674 10.1111/febs.12949

[60] Liu L , Tao Z , Zheng LD , et al. FoxO1 interacts with transcription factor EB and differentially regulates mitochondrial uncoupling proteins via autophagy in adipocytes. Cell Death Discovery. 2016;2(1):16066. 27777789 10.1038/cddiscovery.2016.66 PMC5046220

