# Supplementary figures and images for: Gastrodin induces lysosomal biogenesis and autophagy to prevent the formation of foam cells via AMPK‐FoxO1‐TFEB signalling axis
Source: J Cell Mol Med. 2021 May 10;25(12):5769–81. doi: 10.1111/jcmm.16600 (PMC8184689; doi:10.1111/jcmm.16600)

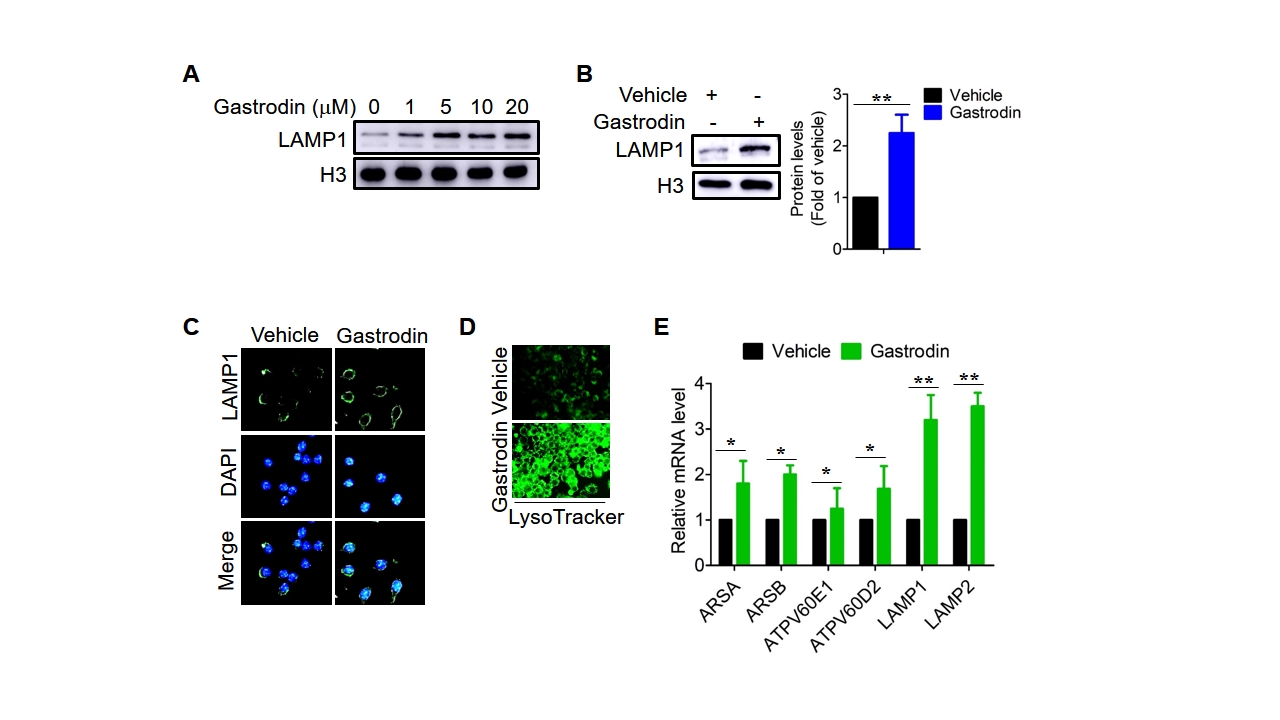

Supplement: Supplementary file 1 — Fig S1 [file JCMM-25-5769-s006.jpg]

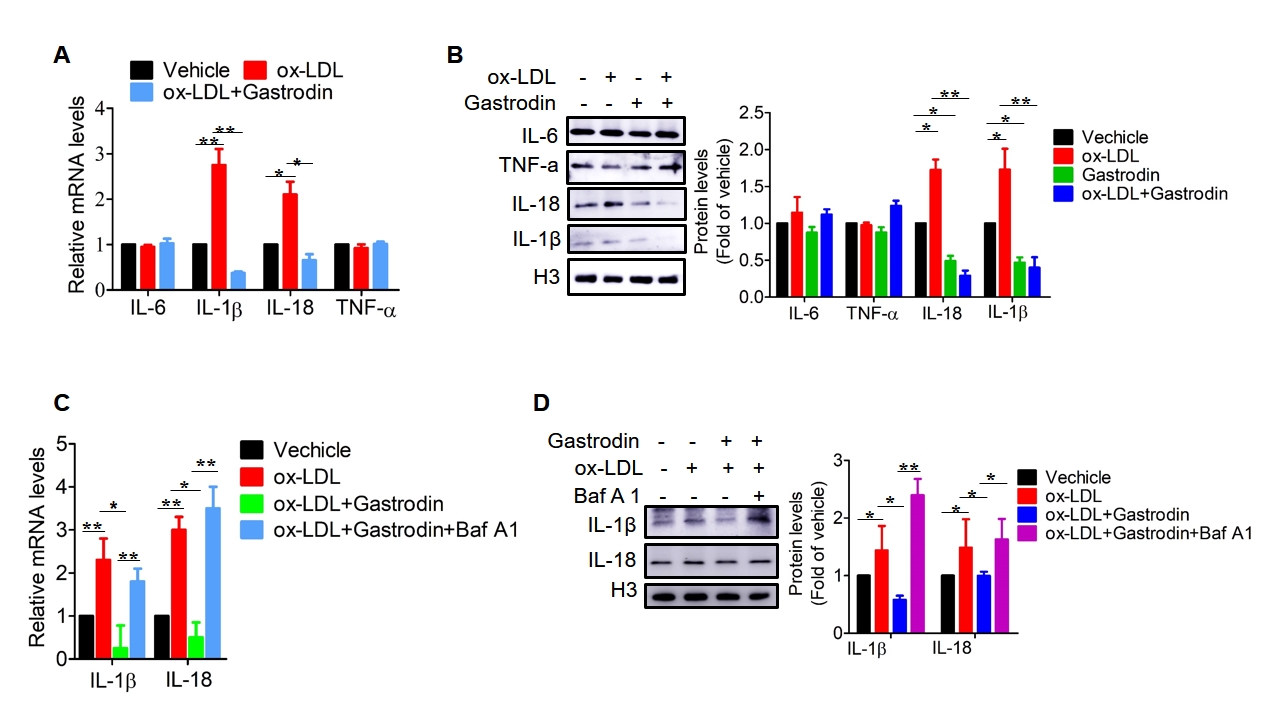

Supplement: Supplementary file 2 — Fig S2 [file JCMM-25-5769-s002.jpg]

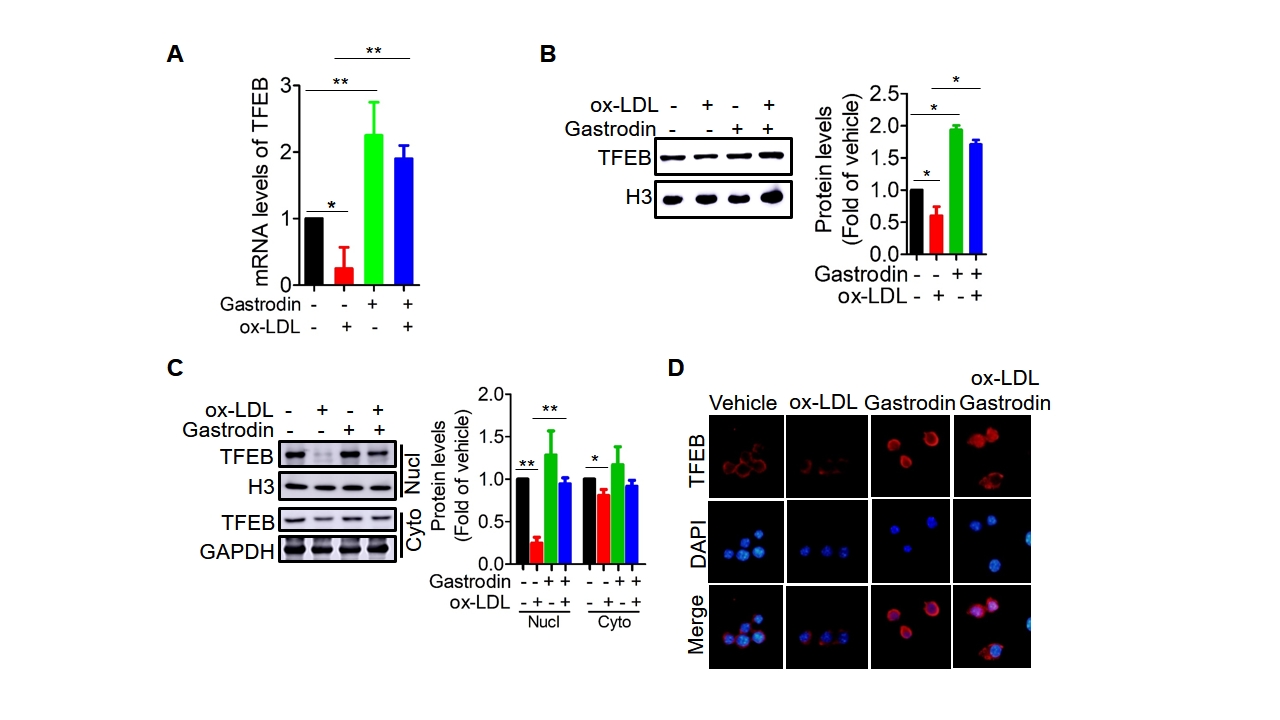

Supplement: Supplementary file 3 — Fig S3 [file JCMM-25-5769-s005.jpg]
