# Supplementary material for: Gastrodin induces lysosomal biogenesis and autophagy to prevent the formation of foam cells via AMPK‐FoxO1‐TFEB signalling axis
Source: J Cell Mol Med. 2021 May 10;25(12):5769–81. doi: 10.1111/jcmm.16600 (PMC8184689; doi:10.1111/jcmm.16600)
Supplement: Supplementary file 4 — Table S1‐S2 [file JCMM-25-5769-s004.docx]

**Supplementary Table S1. Primer sequences used for RT-PCR**

| **Primer** | **Sequence** |
| --- | --- |
| LC3a | Forward:5′-ACACCCATCGCTGACATCT-3′ |
|  | Reverse:5′-AAGCCGAAGGTTTCTTGG-3′ |
| LC3b | Forward:5′-AACCAAGCCTTCTTCCTCC-3′ |
|  | Reverse:5′-CCGTCTTCATCTCTCTCACTTC-3′ |
| Sqstm1/p62 | Forward:5′-TCAGGAGGAGACGATGACTG-3′ |
|  | Reverse:5′-TGGTGGGAGATGTGGGTAT-3′ |
| Beclin-1 | Forward:5′-GCTGGAGTTGGATGACGAA-3′ |
|  | Reverse:5′-TGTGGAAGGTGGCATTGAA-3′ |
| Atg5 | Forward:5′-TCTACACTGTCCATCCAAGG-3′ |
|  | Reverse:5′-TGGTCAAATCTGTCATTCTG-3′ |
| β-actin | Forward:5′-TGGCACCACACCTTCTACA-3′ |
|  | Reverse:5′-TACGACCAGAGGCATACAGG-3′ |
| TFEB | Forward:5′-GCATCAGAAGGTTCGGGAGTA-3′ |
|  | Reverse:5′-ATGGGACTGTTGGGAGCACT-3′ |
| ARSA | Forward:5′-TCTATGTGCCTGTGTCTCTGTG-3′ |
|  | Reverse:5′-CCACTCCAAGATGCCACTT-3′ |
| ARSB | Forward:5′-TGAAGACTATTACACCCACGAGG-3′ |
|  | Reverse:5′-GAAAGCAAGGTAGAGGAACAGTG-3′ |
| ATP6V02D | Forward:5′-GCAAAGCCAGCCTCCTAACT-3′ |
|  | Reverse:5′-TCTTCCTCATCTCCGTGTCA-3′ |
| Lamp1 | Forward:5′-AGTGGGAGTTGCGGTATCA-3′ |
|  | Reverse:5′-TTGGAGATGCTGAATGTGG-3′ |
| Lamp2 | Forward:5′-AAGAAGACCAAACTCCCACC-3′ |
|  | Reverse:5′-GGTAGCCAGCAGACAGGTAGTA-3′ |
| CD36 | Forward:5′-CTTGAAGAAGGAACCACTGCT-3′ |
|  | Reverse:5′-CGAACTCTGTATGTGTAAGGACCT-3′ |
| ABCA1 | Forward:5′-CATCCTACAGTGCTTCCTCATTAG-3′ |
|  | Reverse:5′-CGAAATACTCACAGCCGAATC-3′ |
| ABCG1 | Forward:5′-TGCCTCACCTCACTGTTCA-3′ |
|  | Reverse:5′-TCTTTGACCATCTCTCGTCTG-3′ |
| SR-A | Forward:5′-GAACAACATCACCAACGACC-3′ |
|  | Reverse:5′-GACCAGTTTGTCCAGTAAGCC-3′ |
| IL-6 | Forward:5′-GACTTCCATCCAGTTGCCTT-3′ |
|  | Reverse:5′-CAGGTCTGTTGGGAGTGGTATC-3′ |
| IL-1β | Forward:5′-CTTCAGGCAGGCAGTATCAC-3′ |
|  | Reverse:5′-GCAGGTTATCATCATCATCCC-3′ |
| TNF-α | Forward:5′-CGTCGTAGCAAACCACCAA-3′ |
|  | Reverse:5′-GAGAACCTGGGAGTAGACAAGG-3′ |
| IL-18 | Forward:5′-GCTGTGACCCTCTCTGTGAA-3′ |
|  | Reverse:5′-CATCTTGTTGTGTCCTGGAAC-3′ |
| FoxO1 | Forward:5′-AGAGCGTGCCCTACTTCAA-3′ |
|  | Reverse:5′- CTTTCCAGTTCCTTCATTCTGC-3′ |

**Supplementary Table S2. RNA oligos used for shRNAs**

| **Mouse Gene** | **Oligos** |
| --- | --- |
| TFEB | 5’-TTCTCCGAACGTGTCACGT-3’ |
| FoxO1 | 5’-GCGGGCTGG AAGAATTCAATT-3’ |
|  | 5’-GGAGACTTTCTCCCATCATGA-3’ |
|  | 5’-GCATGTTTATTGAGCGCTTGG-3’ |
| Control shRNA | 5’-TTCTCCGAACGTGTCACGT-3’ |
