# Supplementary material for: Gastrodin induces lysosomal biogenesis and autophagy to prevent the formation of foam cells via AMPK‐FoxO1‐TFEB signalling axis
Source: J Cell Mol Med. 2021 May 10;25(12):5769–81. doi: 10.1111/jcmm.16600 (PMC8184689; doi:10.1111/jcmm.16600)

Raw Data

Figure 1G

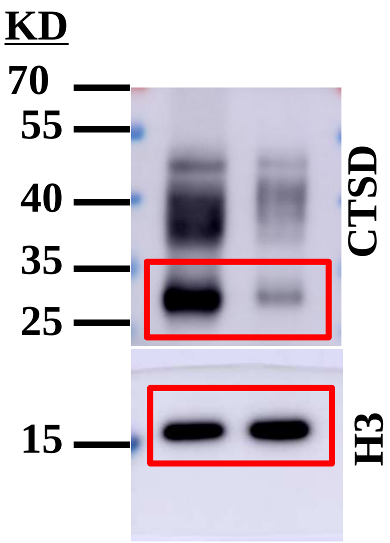

Figure 2E

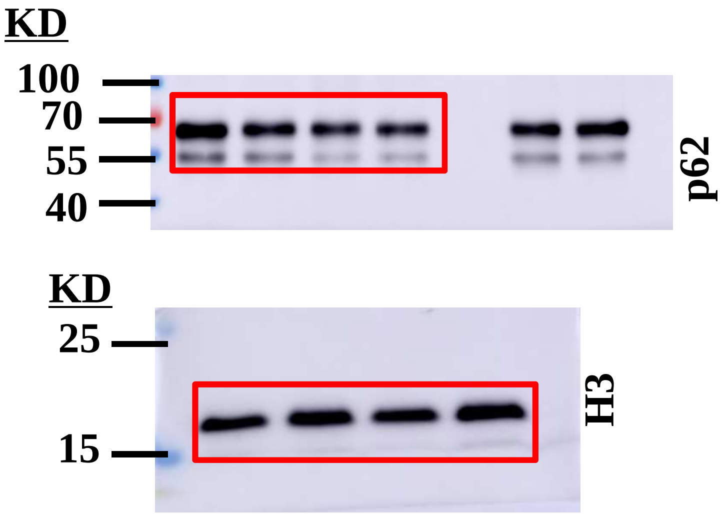

Figure 2A

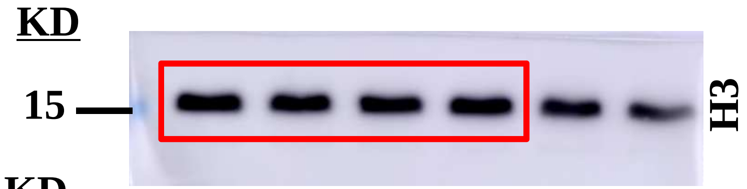

Figure 2D

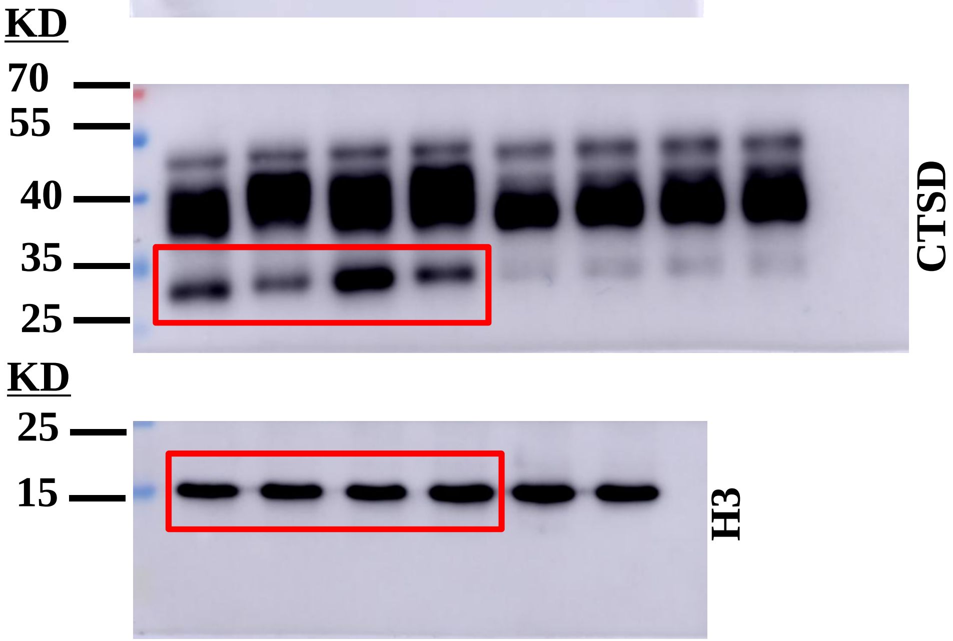

Figure 2F

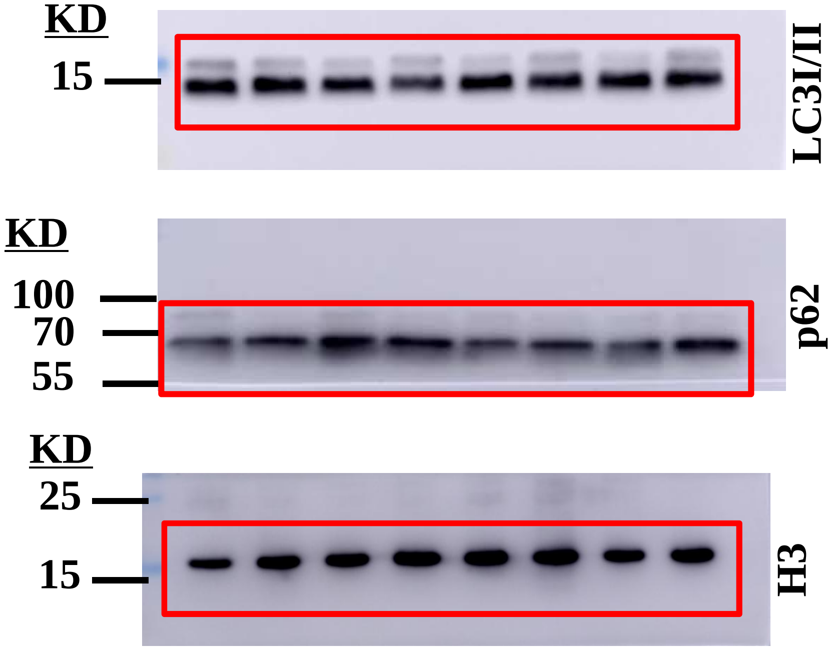

Figure 3E

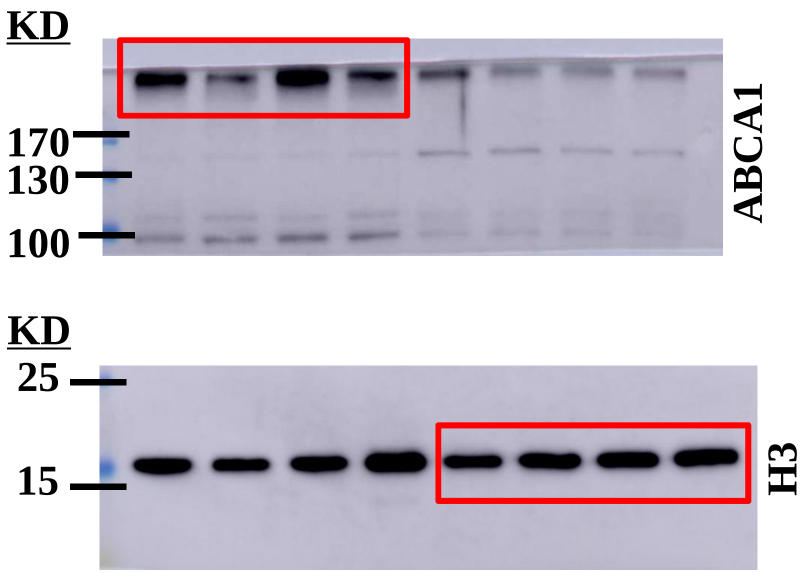

Figure 4C and 6C

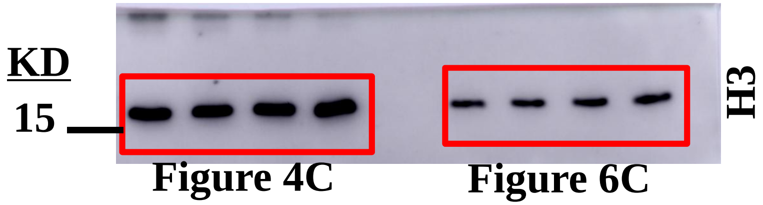

Figure 4 F

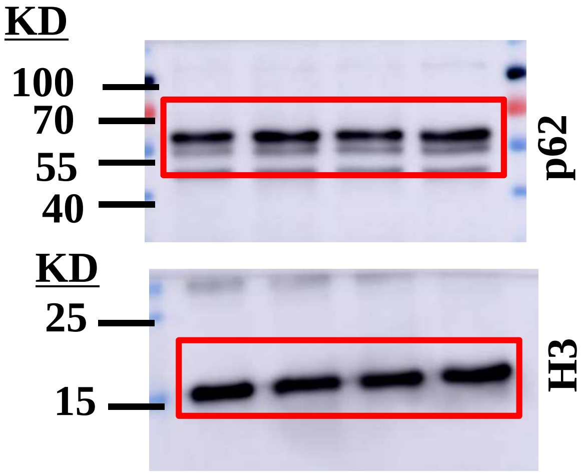

Figure 4B

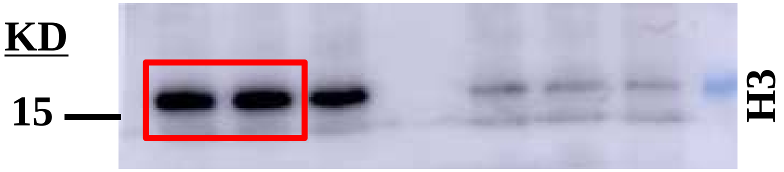

Figure 5A

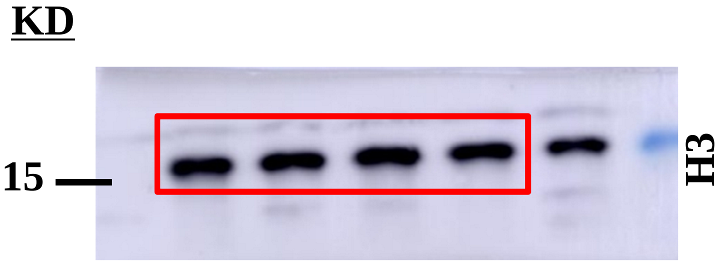

Figure S2B

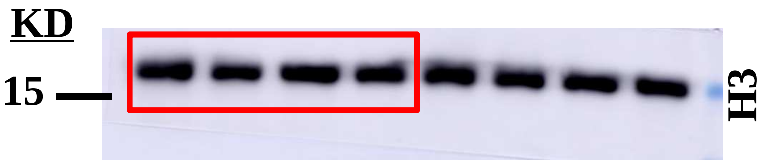

Figure 6A

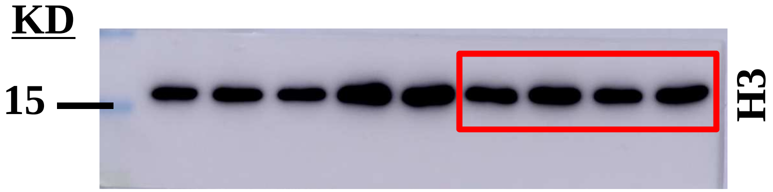

Figure S3B

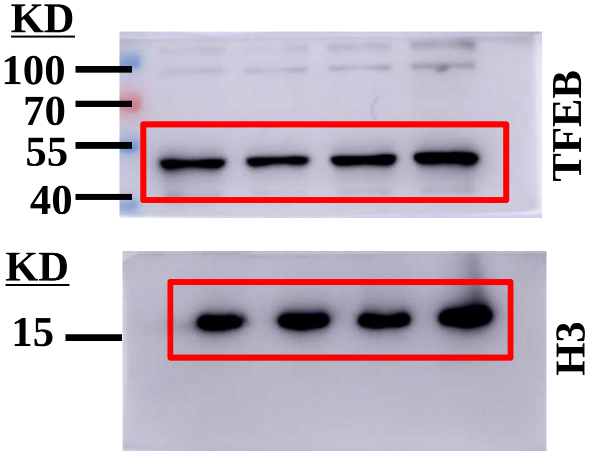

Supplement: Supplementary file 5 — Supplementary Material [file JCMM-25-5769-s003.pdf]
