# Supplementary material for: Gastrodin induces lysosomal biogenesis and autophagy to prevent the formation of foam cells via AMPK‐FoxO1‐TFEB signalling axis
Source: J Cell Mol Med. 2021 May 10;25(12):5769–81. doi: 10.1111/jcmm.16600 (PMC8184689; doi:10.1111/jcmm.16600)
Supplement: Supplementary file 6 — Supplementary Material [file JCMM-25-5769-s001.docx]

**Supplemental Figure** **S1. Gastrodin induces** **lysosomal biogenesis in macrophages.**

(A) Gastrodin increased lysosomal biogenesis in a concentration-dependent manner. Macrophages were treated with different concentration of gastrodin for 24h, and the protein level of LAMP1 was determined by western blotting. (B and C) The expression of LAMP1 was increased in macrophages treated with 20μM of gastrodin. Protein level of LAMP1 was determined by western blotting (B) and immunofluorescence (C). (D) Gastrodin enhanced LysoTracker staining. Macrophages were treated with gastrodin (20μM) for 24h and stained with LysoTracker. (E) The mRNA levels of lysosome biogenesis-related genes were determined by RT-PCR. *P < 0.05; **P < 0.01. Results are means ± SD of three independent experiments. The value represents fold of vehicle.

**Supplemental Figure S2. Gastrodin decreases inflammation through autophagy.**

(A and B) Gastrodin decreased the expressions of pro-inflammation cytokines (IL-1β and IL-18), including mRNA levels (A) and protein levels (B), but not IL-6 and TNF-α. (C and D) Macrophages were pretreated with Baf A1 (25nM) and then treated with gastrodin and ox-LDL. Measurement of the mRNA levels (C) and protein levels (D) of IL-1β and IL-18. *P < 0.05; **P < 0.01. Results are presented as mean ± SD of three independent experiments. The value represents fold of vehicle.

**Supplemental Figure S3. Gastrodin promotes TFEB activation in the foam cells.**

(A and B) Gastrodin upregulated the expression of TFEB in the foam cells. The mRNA level (A) and protein level (B) of TFEB were determined. (C and D) Gastrodin promoted the nuclear translocation of TFEB in the foam cells. (C) Western blotting analysis of cytoplasmic and nuclear fractions of TFEB. (D) Images of the subcellular locations of TFEB. *P < 0.05; **P < 0.01. Results are presented as mean ± SD of three independent experiments. The value represents fold of vehicle. Cyto, Cytoplasm; Nucl, Nucleus.
